# FUS Microphase Separation: Regulation by Nucleic Acid Polymers and DNA Repair Proteins

**DOI:** 10.3390/ijms232113200

**Published:** 2022-10-30

**Authors:** Maria V. Sukhanova, Rashid O. Anarbaev, Ekaterina A. Maltseva, David Pastré, Olga I. Lavrik

**Affiliations:** 1 Institute of Chemical Biology and Fundamental Medicine (ICBFM), SB RAS, 630090 Novosibirsk, Russia; 2 SABNP, University of Evry, INSERM U1204, Université Paris-Saclay, 91025 Evry, France

**Keywords:** FUS, nucleic acid polymer, microphase separation, DNA repair protein

## Abstract

Fused in sarcoma (FUS) is involved in the regulation of RNA and DNA metabolism. FUS participates in the formation of biomolecular condensates driven by phase transition. FUS is prone to self-aggregation and tends to undergo phase transition both with or without nucleic acid polymers. Using dynamic light scattering and fluorescence microscopy, we examined the formation of FUS high-order structures or FUS-rich microphases induced by the presence of RNA, poly(ADP-ribose), ssDNA, or dsDNA and evaluated effects of some nucleic-acid-binding proteins on the phase behavior of FUS–nucleic acid systems. Formation and stability of FUS-rich microphases only partially correlated with FUS’s affinity for a nucleic acid polymer. Some proteins—which directly interact with PAR, RNA, ssDNA, and dsDNA and are possible components of FUS-enriched cellular condensates—disrupted the nucleic-acid-induced assembly of FUS-rich microphases. We found that XRCC1, a DNA repair factor, underwent a microphase separation and formed own microdroplets and coassemblies with FUS in the presence of poly(ADP-ribose). These results probably indicated an important role of nucleic-acid-binding proteins in the regulation of FUS-dependent formation of condensates and imply the possibility of the formation of XRCC1-dependent phase-separated condensates in the cell.

## 1. Introduction

Human proteins containing low-complexity regions are a large family of proteins that can undergo phase separation and participate in the organization of membraneless compartments in vivo [1]. One of them is a multifunctional RNA/DNA-binding protein called fused in sarcoma (FUS, also known as FUS/TLS) [2]. FUS belongs to the highly con-served FET family of RNA-binding proteins, and its biological functions are mainly associated with the metabolism of mRNA; e.g., pre-mRNA splicing, mRNA transport, and local mRNA translation in neurons [3]. 

FUS is an intrinsically disordered protein composed of a N-terminal low-complexity domain (LCD) with prion-like sequences, three arginine/glycine/glycine (RGG)-rich regions, a conserved RNA recognition motif (RRM), a zinc finger (ZnF) motif, and a pro-line-tyrosine nuclear localization signal (PY-NLS) at the C terminus [4]. The LCD, also called the prion-like domain, has been shown to promote FUS self-assembly into higher-order structures, which contributes to either liquid–solid or liquid–liquid phase separation (LSPS or LLPS) of FUS either alone or in combination with other proteins and/or nucleic acids [5,6,7,8,9,10]. 

It has been proposed that the capacity of FUS for LLPS, also known as biomolecular condensation, helps to generate transient membraneless organelles such as neuronal ribonucleoprotein granules, stress granules, and the P-bodies in the cytoplasm, as well as to organize paraspeckles and DNA repair condensates in the nucleus [11,12,13,14,15]. Although it has been reported that FUS takes part in the formation of DNA double-strand break repair foci with liquid-like properties [15], there is currently no clear evidence that double-stranded DNA (dsDNA) (or single-stranded DNA; ssDNA) and FUS form condensates in the cell. At the same time, both RNA and poly(ADP-ribose) (PAR) have been shown to regulate FUS condensation and are considered some of the critical factors driving FUS LLPS in the cell [8,14,16,17]. The interaction of FUS and PAR is of special interest for research on PAR-dependent DNA repair capacity because this process does not usually involve RNA-binding proteins [18,19]. In the nucleus, PAR is synthesized by PARP1 or by PARP2, which are enzymes from the diphtheria toxin-like family of ADP-ribosyltransferases, also known as poly(ADP-ribose) polymerases (PARPs) [20]. PARP1 and PARP2 are activated upon their binding to damaged DNA, use NAD^+^ as a substrate, and mainly catalyze the transfer of ADP-ribose units from NAD^+^ onto their own amino acid residues, resulting in their own poly(ADP-ribosyl)ation (PARylation) [20,21]. Thus, ADP-ribose units are building blocks of PAR, which is a nucleic-acid-like polymer that shares several features with single-stranded RNA (ssRNA) or ssDNA, although PAR is mostly a branched polymer [22]. PARP1 and PARP2 are primarily known as regulators of base excision repair and DNA single-strand break repair; namely, as proteins that participate in overall coordination of the repair machinery and individual enzymes [23]. Given that FUS is prone to self-assembly and undergoes cocondensation with other proteins or nucleic acids [5,6,14,16,24], FUS is thought to drive the formation of repair condensates, and PAR acts as a condensate-forming scaffold molecule in the process [8,14,25]. Our previous in vitro studies indicated that the recruitment of FUS to DNA damage sites—via binding to the PAR synthesized during PARP1 activation and autoPARylation—gives rise to large molecular assemblies containing damaged DNA, PARylated PARP1, and FUS [25]. In vitro, FUS has also been reported to form liquid-like condensates with various nucleic acid polymers such as long lambda phage dsDNA and ssDNA molecules, PAR, poly(U), or short heterogeneous RNAs [8,9,16,25,26,27,28].

Although the assembly of FUS high-order structures and FUS LLPS in the presence of RNA, DNA, or PAR have been extensively characterized in vitro [8,9,10,16,26,27,28], the influence of other proteins related to DNA-, RNA-, and PAR-dependent metabolic processes has not been investigated yet.

In this work, we attempted to elucidate how different biomolecules can contribute to the assembly of FUS high-order structures. Using dynamic light scattering (DLS) and fluorescence microscopy, we characterized in detail nucleic-acid-induced FUS microphase separation in a wide range of nucleic acid polymer concentrations and compared the properties of PAR, RNA, ssDNA, and dsDNA as molecular “seeds” for the promotion of the assembly of the protein structures. To this aim, we carried out our experiments under conditions in which FUS was primarily monomeric and its microphase separation was promoted in the presence of nucleic acids. We estimated the range of the molar FUS-to-nucleotide ratio that was favorable for the stability of the protein phase-separated state. We observed that individual proteins that directly interacted with PAR, RNA, ssDNA, and/or dsDNA disrupted the FUS–nucleic acid droplet assemblies. We also found that XRCC1 (a key repair factor for DNA single-strand breaks) underwent microphase separation (forming its own protein-rich microphases) and coassembly with FUS in the presence of PAR. The dependence of PAR-, RNA-, and DNA-induced FUS microphase separation on nucleic-acid-binding proteins points to a potential role of the latter in the regulation of formation of FUS-related biological condensates; this function requires further investigation.

## 2. Results

### 2.1. Nucleic Acid Polymers Promote the Assembly of FUS Monomer into High-Order Structures

FUS has long been thought to take part in RNA metabolism [29,30]. FUS binds to RNA and functions in the regulation of transcription, alternative splicing, and nuclear–cytoplasmic mRNA transport [3]. Nevertheless, FUS is also able to bind PAR and different types of DNA and has been implicated in processes involving PAR or DNA such as DNA repair [8,9,14,15,25,31,32,33,34,35]. Recently, much attention was given to the potential involvement of other biopolymers such as PAR, DNA, or RNA in the promotion of FUS LLPS or condensation in vitro and in vivo [8,9,14,16,25,26,27,28,36].

**Figure 1:**
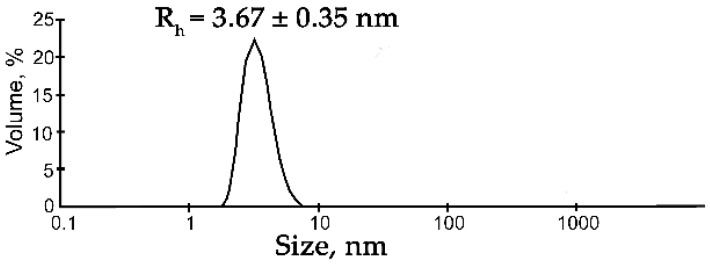
DLS analysis of the FUS solution. Typical volume-weighted size distributions for FUS. The profile was obtained by means of experimental autocorrelation functions in the Zetasizer Nano ZS software. The average hydrodynamic radius (R_h_) computed from the distributions is presented as well. R_h_ is the average R_h_ value estimated from at least three DLS experiments (Table S1).

Here, we compared the propensity of FUS for phase separation in the presence of different types of nucleic acid polymers. For this purpose, we employed DLS to assess the hydrodynamic size of protein assemblies arising in the presence of protein-free PAR (heterogeneous in length from 4 to more than 30 nt), an RNA transcript (~3000 nt), ssDNA (29 nt), or dsDNA (30 bp; Table 1). FUS was shown to be prone to LLPS and spontaneous self-assembly generally in the micromolar range even in the absence of nucleic acids [6,24,37]. Therefore, we tested the buffer conditions at which FUS showed a low tendency toward self-assembly/aggregation over time by varying the buffering agents and the concentrations of NaCl and urea and used dynamic light scattering (DLS) to monitor the aggregation state of FUS in solution. According to the DLS analysis, the size of FUS particles across the 0.1–10,000.0 nm range was determined at various concentrations of the protein (5–10 µM), urea (0.200–1 M), and NaCl (100–200 mM) and in different buffers (Tris-HCl pH 7.5 or HEPES-NaOH pH 7.5). FUS (5–10 µM) was found to be mainly a monomer, and the amount of oligomers was quite small in the presence of urea at 300 mM and NaCl at 200 mM (Figure 1). 

**Figure 2:**
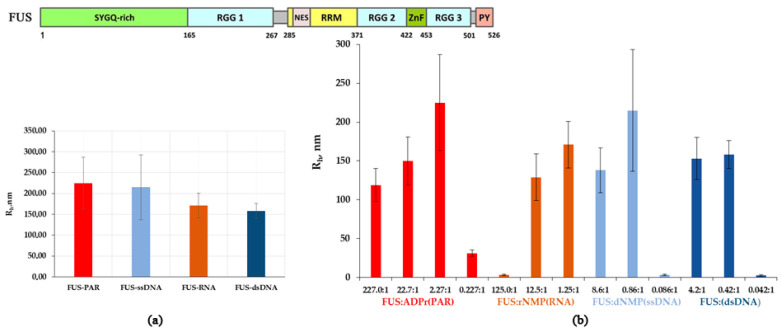
Effect of the different concentrations of RNA(DNA) nucleotides and ADPr on the assembly of FUS high-order structures. (**a**) R_h_ of FUS–nucleic acid mixtures presented as a function of the FUS-to-nucleotide molar ratio (Table S1). R_h_ is the average R_h_ value estimated from at least three DLS experiments. (**b**) Comparison of maximum hydrodynamic radii (R_h_, nm) for FUS-PAR, FUS-ssDNA, FUS-RNA, or FUS-dsDNA mixtures (Table S1). R_h_ is the average R_h_ value estimated at a [FUS]:[ADPr] ratio of 2.27:1, [FUS]:[rNMP] ratio of 1.25:1, [FUS]:[dNMP(ssDNA)] ratio of 0.86:1, and [FUS]:[dNMP(dsDNA)] ratio of 0.42:1; upper panel: a schematic diagram of the domain structure of the FUS wild type. SYGQ-rich: serine/tyrosine/glycine/glutamine-rich, low complexity domain (LCD); RGG(1–3): arginine/glycine/glycine-rich regions; RMM: RNA recognition motif; ZnF: zinc finger motif; PY: proline-tyrosine nuclear localization signal at the C terminus.

Under these conditions, FUS particles with a hydrodynamic radius (R_h_) of 3.67 ± 0.35 nm were detectable, which corresponded to the monomer of the protein (for a spherical globular protein with a molecular weight of 54.4 kDa, the theoretical radius is 3.26 nm; Figure 1, Table S1). For this reason, we chose these buffering conditions for further DLS analysis of the assembly of FUS. Recently, RNA and ssDNA were found to enhance FUS phase separation at low concentrations and to trigger the reentrant phase transition at high concentrations [17,27]. Therefore, we monitored changes in the FUS hydrodynamic size at various concentrations of ssDNA, dsDNA, RNA, or PAR (Figure 2 and Figure S1, Table S1). In the presence of DNA, RNA, or PAR, the formation of large particles with a radius of 119 to 225 nm was observed; the FUS structures’ maximal size could be ranked in the following order: FUS–dsDNA ≤ FUS–mRNA < FUS–ssDNA ≤ FUS–PAR (Figure 2a).

We found that all nucleic acids induced the assembly of FUS into high-order structures or protein-rich microphases that were detected within a certain range of the nucleic acid concentrations (Figure 2b). Upon the addition of a nucleic acid, FUS high-order assemblies arose at different FUS:nucleotide molar ratios in the range of 227:1 to 2.2:1.0 for PAR ([FUS]:[ADP-ribose]), 12.5:1.0 to 1.25:1.00 for RNA ([FUS]:[rNMP]), 8.6:1.0 to 0.86:1.00 for ssDNA ([FUS]:[dNMP]), and 4.2:1.0 to 0.42:1.00 for dsDNA ([FUS]:[dNMP]) (Figure 2a). In the case of PAR, particles with an R_h_ of 119 to 225 nm formed in a broad range of the [protein]:[ADPr] ratio (from~200:1 to~2.2:1.0), whereas in the cases of RNA, ssDNA, and dsDNA, large particles were found in a narrow range (12.5:1.0 to 0.42:1.00) of the [FUS]:[nucleotide] ratio (Figure 2b). On the one hand, our results showed similar patterns of FUS microphase separation induced by the presence of different nucleic acids. For example, if the [FUS]-to-[nucleotide] ratio was ≥1:1, then large particles (R_h_ ≈ 120–225 nm) formed, but nucleic acids readily disrupted assemblies when present in a large molar excess over FUS (Figure 2b). Under these conditions, large assemblies dissociated, and particles with smaller R_h_ (~31–3 nm) were detectable. Nevertheless, microphase separation started at a relatively high FUS-to-PAR ratio (~200:1), whereas the formation of large assemblies was observed at a lower FUS-to-rNMP(dNMP) ratio (~10:1) (Figure 2b). Thus, we were able to determine the optimal nucleic acid concentrations at which the formation of relatively stable FUS high-order structures or protein-rich microphases were detected. Consequently, the FUS-rich microphase separated state strongly depended on the FUS-to-nucleotide ratio, which was different for each type of nucleic acid polymer.

**Figure 3:**
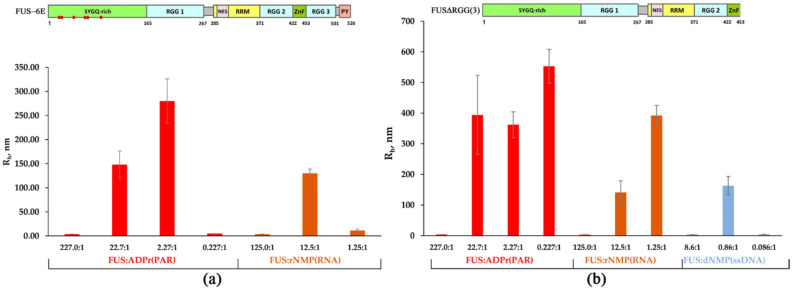
Effect of the mutations in LCD and deletion of RGG(2,3) domains on the formation of FUS-rich microphases induced by the presence of PAR, RNA, or ssDNA. (**a**) The hydrodynamic radius (R_h_, nm) for FUS-6E mutant as determined in the presence of different concentrations of rNMP(RNA) and ADPr (PAR). R_h_ of FUS-6E–RNA(or PAR) mixtures is presented as a function of the FUS-6E-to-nucleotide molar ratio. R_h_ is the average R_h_ value estimated from at least three DLS experiments (Table S2); upper panel: a schematic diagram of domain structure of FUS-6E; (•) phosphomimetic mutations are amino acid substitutions (Ser/Tyr to Glu). (**b**) The hydrodynamic radius (R_h_, nm) for FUSΔRGG3 as determined in the presence of different concentrations of RNA(ssDNA) nucleotides and ADPr. R_h_ of FUSΔRGG3–nucleic acid mixtures is presented as a function of the FUS-to-nucleotide molar ratio. R_h_ is the average R_h_ value estimated from at least three DLS experiments (Table S3); upper panel: a schematic diagram of domain structure of FUS∆RGG(3).

The phase separation property of FUS is based on its ability to self-assemble and/or interaction with various biopolymers, including RNA, PAR, DNA [2,6,8,9,14,15,16,17,24]. Thus, intermolecular interactions play a significant role in driving FUS phase separation [38]. Some studies have identified the N-terminal LCD and RGG(2,3) domains as critical for the regulation of the phase behavior of FUS; LCD is not involved in binding of nucleic acids but mediates FUS self-assembly [8,25,39,40]. In turn, RGG(2,3) domains possess nucleic-acid-binding activity and are involved in PAR- or RNA-dependent regulation of a high-order assembly of FUS [8,25,39], although little is known about how the domains affect FUS phase separation in the presence of DNA [9]. Therefore, we tested the effect of FUS mutations in LCD or RGG(2,3) domains in the formation of nucleic-acid-induced FUS-rich microphases under our experimental conditions. For this purpose, we utilized FUS phosphomimetic mutants containing S26E, S30E, T68E, S84E, S87E, and S117E or T7E, T11E, T19E, S26E, S30E, S42E, S61E, T68E, S84E, S87E, S117E, and S131E mutations introduced into the LCD domain (FUS-6E and FUS-12E, respectively); or RGG3- or RGG2,3-deleted FUS mutants (FUSΔRGG(3) and FUSΔRGG(2,3), respectively). The phosphomimetics FUS-6E and FUS-12E with substitutions of serine (or threonine) with glutamic acid residues in the LCD domain have a lower self-association propensity [41], whereas the mutants FUSΔRGG(3) and FUSΔRGG(2,3) show lower RNA and PAR binding as compared to the wild-type FUS [8,25,39]. At first, we evaluated the assembly of FUS phosphomimetics (FUS-6E or FUS-12E) into high-order structures in the presence of PAR, RNA, ssDNA, or dsDNA (Figure 3a and Figure S2, Table S2).

According to our data, both the FUS-6E and FUS-12E mutants possessed a weaker ability to form high-order assemblies (R_h_ > 100 nm) in the presence of all types of tested nucleic acids (Figure 3a and Figure S2, Table S2). In fact, the FUS-6E mutant partially retained the capacity for microphase separation, which was found to occur only in the presence of PAR or RNA, whereas the FUS-12E mutant was devoid of the ability to form large assemblies but could still form high-molecular-weight oligomers in the presence of PAR (Figure 3a and Figure S2). This implied that LCD indeed was involved in the assembly of FUS-rich microphases in the presence of nucleic acids under these conditions, but this process also depended on the type of nucleic acid because PAR and RNA was more effective in the promotion of the FUS-6E microphase separation than ssDNA and dsDNA (Figure 3a).

Next, we addressed the formation of high-order FUSΔRGG(3) and FUSΔRGG(2,3) structures initiated by the presence of PAR, RNA, ssDNA, or dsDNA (Figure 3b and Figure S3, Table S3). As in the case of FUS-6E and FUS-12E, the assembly of FUS into high-order structures was noticeably affected for both FUS mutants and featured a deletion in the RGG domain(s) (Figure 3b). For FUS∆RGG(3), in contrast to the wild-type protein, phase separation did not occur only in the presence of dsDNA (Table S3); FUS∆RGG(2,3) manifested no ability to form high-molecular-weight oligomers (Rh ≤ 30 nm) and large assemblies (Rh > 100 nm) in the presence of all types of nucleic acids tested here (Figure 3b, Table S3). Therefore, the RGG(2,3) truncation mutant was drastically reduced in its ability to interact with any of the nucleic acid polymers and therefore was quite insensitive to the nucleic acid as an initiator of phase separation. Thus, these mutations in LCD and the deletion of RGG domains strongly impaired nucleic acid-induced assembly of FUS into high-order structures, since FUS-6E and FUS-12E had a reduced ability to self-associate [41], and FUS∆RGG(3) and FUS∆RGG(2,3) seemed to bind with nucleic acids with reduced or very low affinity.

Overall, these experiments suggested that the phase behavior of FUS and its mutants in the presence of a nucleic acid polymer was strongly dependent on the [FUS]-to-[nucleotide] ratio (Figure 2 and Figure 3), and the range of these ratios suitable for the microphase separation depended on the type on nucleic acid and may have been sensitive to the affinity of the protein for the nucleic acid.

### 2.2. Properties of the Binding of FUS to Nucleic Acid Polymers

Some studies have revealed that FUS binds to PAR and RNA with different affinity values, and the same is true for RNA, ssDNA, and dsDNA [26,31]. Considering that PAR, RNA, or DNA molecules play a prominent part in FUS condensation [8,9,14,16,25,26,27,28], we hypothesized that FUS phase separation may directly correlate with properties of the binding of FUS to these nucleic acid polymers. To test this idea, we compared the affinity of FUS for the ssDNA, dsDNA, RNA, and PAR substrates used in the DLS assay. To this end, we conducted fluorescence titration experiments with Cy3-labeled FUS by means of a change in the fluorescence intensity signal as an indicator of protein–nucleic acid complexation. First, we noticed that full-length Cy3-FUS, even at a low concentration (~20 nM), was prone to oligomerization and/or assembly; as a result, a reproducible shape of the titration curve was not obtained (data not shown). The N-terminal LCD mainly promotes FUS’s self-assembly, interaction with other proteins, and phase separation [2,5,6,7,24,41], whereas three RGG-rich regions (named RGG1–3), the RRM, and the ZnF are involved in the interaction with nucleic acids [8,16,25,27,39,40]. Therefore, in the binding assays, we decided to use the N-terminally truncated FUS (aa 164–526); i.e., FUS∆LCD, which contained the nucleic-acid-binding motifs RGG1–3, RRM, and ZnF (Figure 2, upper panel). The complexation of Cy3-labeled FUS∆LCD with nucleic acids was analyzed by means of PAR, RNA, ssDNA, or dsDNA (Table 2). The apparent binding affinity (K_d_,_app_) was estimated by monitoring fluorescence intensity changes in the Cy3-labeled protein in the presence of various PAR, RNA, ssDNA, or dsDNA concentrations (Table 2, Figure S4). Because the nucleic acid substrates in question were heterogeneous in chain length and structure and differed in total charge, their concentrations employed during the titration experiments are presented as molar concentrations of ADPr, rNMP, or dNMP for PAR, RNA, or DNA, respectively.

A direct comparison of FUS∆LCD’s affinity values for PAR, RNA, ssDNA, and dsDNA revealed clear differences in K_d,app_; this protein manifested the following ranking in binding affinity: ADPr > rNMP >> dNMP(ssDNA) > dNMP(dsDNA), with the range of the binding constant spanning one order of magnitude. These results further supported the findings of other studies that observed that full-length FUS showed a preference for the binding to RNA over ssDNA(dsDNA) as well as PAR over RNA [26,31]. As presented in Table 2, FUS∆LCD had a nearly 1.6-fold stronger K_d,app_ for ADPr (~114 nM) than for rNMP (~183 nM), showing that FUS had a preference for the binding to PAR over RNA. In contrast to ADPr, FUSΔLCD possessed a nearly 10.0- to 22.0-fold weaker affinity for dNMP(ssDNA) and dNMP (dsDNA) with a K_d,app_ of ~1.1 and~2.5 µM, respectively (Table 2).

Thus, the strong affinity of FUS for ADPr could explain the PAR-induced microphase separation in the wide range of the [FUS]-to-[ADPr] ratio of ~ 200:1 to 2:1 (Figure 2). In contrast, FUS had a weak affinity for dNMP, and the formation of large assemblies was registered within a narrow range of the [FUS]-to-[dNMP] ratio of ~10:1 to 1:2 (Figure 2). FUS microphase separation also appeared to depend on the length or branching of a nucleic acid. This was because we also observed RNA-initiated large assemblies within a narrow range of the [FUS]-to-[rNMP] ratio of ~10:1 to 1:1 (Figure 2), even though FUSΔLCD showed strong affinity for rNMP, which was close to that for ADPr (Table 2). Thus, FUS microphase separation was dependent not only on the protein’s affinity to a nucleic acid polymer but also on the structure of the latter. Therefore, nucleic acid polymer flexibility, length, and branching along with the binding affinity of FUS for the nucleic acid may exert a substantial influence on the phase behavior of FUS.

### 2.3. DNA Repair Proteins Disrupt the Formation of FUS–Nucleic Acid Microphases In Vitro

**Figure 4:**
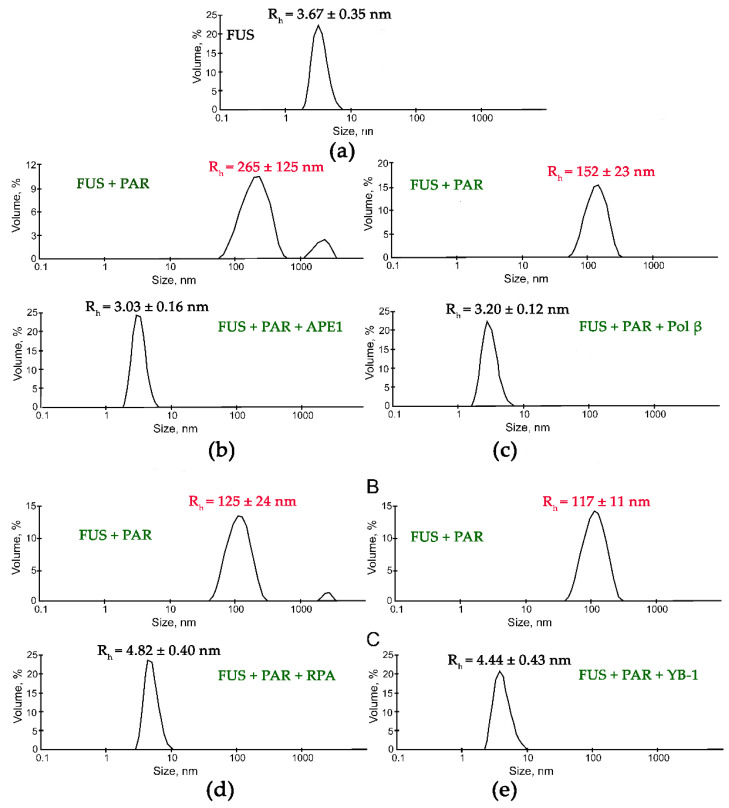
DNA repair proteins disrupt the FUS-PAR microphases. Typical volume-weighted size distributions for FUS (**a**) and a FUS–PAR mixture before and after the addition of APE1 (**b**), Polβ (**c**), RPA (**d**), or YB-1 (**e**). The profiles were obtained by means of experimental autocorrelation functions in the Zetasizer Nano ZS software. The average hydrodynamic radii (R_h_) computed from the distributions are presented as well. R_h_ is the average R_h_ value estimated from at least three DLS experiments. FUS–PAR high-order structure assays in the presence of PAR-binding proteins were performed in reaction mixtures consisting of: (**a**) 10 μM FUS; (**b**) 10 μM FUS, 0.44 µM ADPr(PAR), and 5.9 µM APE1; (**c**) 10 μM FUS, 0.88 µM ADPr(PAR), and 10 µM Polβ; (**d**) 10 μM FUS, 0.44 µM ADPr(PAR), and 1 µM RPA; (**e**) 10 μM FUS, 0.88 µM ADPr(PAR), and 2.4 µM YB-1. The R_h_ values were measured directly before and after 1-min incubation of FUS-PAR mixture with proteins.

**Figure 5:**
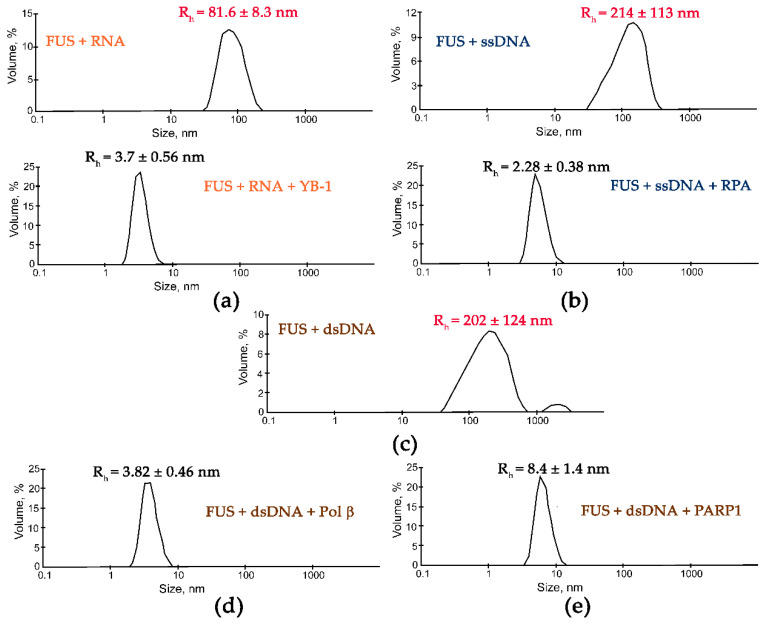
DNA repair proteins disrupt the FUS-RNA or FUS-DNA microphases. Typical volume-weighted size distributions for mixtures FUS–RNA (**a**), FUS–ssDNA (**b**), and FUS–dsDNA (**c**) before and after the addition of Yb-1 (**a**), RPA (**b**), Polβ (**d**), or PARP1 (**e**). The profiles were obtained by means of experimental autocorrelation functions in the Zetasizer Nano ZS software. The average hydrodynamic radii (R_h_) computed from the distributions are presented as well. R_h_ is the average R_h_ value estimated from at least three DLS experiments. FUS–nucleic acid high-order structure assays in the presence of RNA-/ssDNA-/dsDNA-binding proteins were performed in reaction mixtures consisting of: (**a**) 10 μM FUS, 0.77 µM rNMP(RNA), and 2 µM YB-1; (**b**) 10 μM FUS, 5.5 µM dNMP(ssDNA), and 2.2 µM RPA; (**с**) 10 μM FUS and 7 µM dNMP(dsDNA); (**d**) 10 μM FUS, 7 µM dNMP(dsDNA), and 2.5 µM Polβ; (**e**) 10 μM FUS, 8.9 µM dNMP(dsDNA), and 2.5 µM PARP1. The R_h_ values were measured directly before and after 1 min incubation of FUS–nucleic mixture with proteins.

Although the presence of PAR, RNA, or DNA strongly stimulates FUS condensation, little is known regarding the effects of other nucleic-acid-binding proteins on the formation of high-order FUS structures. Indeed, other proteins with DNA-, PAR-, or RNA-binding activity can affect the FUS condensation or LLPS in the cell. To gain insight into the impact of other nucleic-acid-binding proteins on the nucleic-acid-induced assembly of FUS into high-order structures, we chose replication protein A (RPA), Y-box binding protein 1 (YB-1), apurinic/apyrimidinic endonuclease 1 (APE1), DNA polymerase β (Polβ), X-ray repair cross-complementing 1 (XRCC1), and PARP1. RPA and YB-1 most often bind ssDNA and RNA, respectively, and have also been reported to interact with PAR [42,43,44,45]. Polβ and PARP1 participate in base excision repair/DNA single-strand break repair and can interact with dsDNA [23,46]; in particular, the damaged 30 bp DNA containing one nucleotide gap (dsDNA, Table 1) is a base excision repair DNA intermediate and a substrate for Polβ’s gap-filling activity and for PARP1 binding [23,46,47]. XRCC1 (a DNA single-strand break repair factor) and APE1 (a base excision repair enzyme) not only possess a DNA-binding activity but also bind with high affinity to PAR [48,49,50]. To determine whether these proteins influence the FUS microphase separation, we carried out a DLS analysis of assembly of FUS into high-order structures in the presence of ssDNA and RPA; dsDNA and Polβ or PARP1; RNA and YB-1; or PAR and APE1, YB-1, RPA, or XRCC1 using the [FUS]-to-[nucleotide] ratio needed to observe the stable formation of FUS–nucleic acid microphases. Under our experimental conditions, all the tested proteins except for XRCC1 disrupted the large FUS assemblies induced by the presence of PAR, RNA, ssDNA, or dsDNA. Upon the addition of PAR, RNA, ssDNA, or dsDNA, FUS particles in the 81.6–265.0 nm range were detectable (Figure 4 and Figure 5).

Subsequent addition of another protein (APE1, YB-1, RPA, PARP1, or Polβ) to the FUS–nucleic acid solutions resulted in the dissociation of the assemblies accompanied by a reduction in the particle size down to 3.3–8.4 nm, which matched oligomers of these proteins and/or their complexes with nucleic acids (Figure 4 and Figure 5).

These data suggested that these proteins competed with FUS for nucleic acid binding and/or influenced FUS self-association, thereby impeding the nucleic-acid-induced FUS microphase separation.

**Figure 6:**
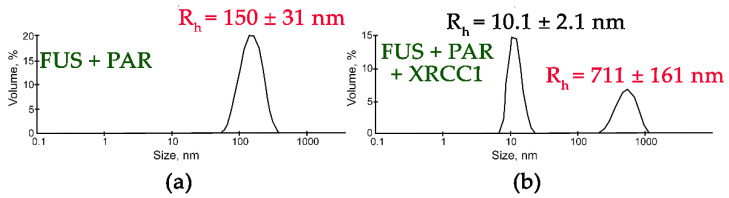
XRCC1 influences the size of FUS-PAR high-order assemblies. Typical volume-weighted size distributions for FUS–PAR before (**a**) and after the addition of XRCC1 (**b**). The profiles were obtained via experimental autocorrelation functions in the Zetasizer Nano ZS software. The average hydrodynamic radii (R_h_) computed from the distributions are presented as well. R_h_ is the average R_h_ value estimated from at least three DLS experiments. FUS–PAR high-order structure assays in the presence of XRCC1 were performed in reaction mixtures consisting of: (**a**) 10 μM FUS and 0.44 µM ADPr(PAR); (**b**) 10 μM FUS, 0.44 µM ADPr(PAR), and 2.1 µM XRCC1. The R_h_ values were measured directly before and after 1 min incubation of FUS-PAR mixture with XRCC1.

In contrast to the results obtained with YB-1, APE1, RPA, PARP1, or Pol β, an analysis of the influence of XRCC1 on FUS assemblies formed in the presence of PAR indicated that two types of particles with an R_h_ in the range of 10 to 711 nm were present (Figure 6). Although particles with a size corresponding to that of FUS–PAR assemblies (average R_h_ ~150 nm) disappeared, larger particles (average R_h_ ~711 nm) formed instead (Figure 6). These large particles may have represented higher-order FUS–XRCC1–PAR, FUS–PAR, and/or XRCC1–PAR assemblies.

XRCC1 is a disordered protein that serves as a scaffold for the recruitment of single-strand break/base excision repair proteins such as APE1, Polβ, and DNA ligase III [51]. Moreover, XRCC1 has been found to accumulate at DNA breaks through interaction with the PAR synthesized due to PARP1 (or PARP2) activation after DNA damage [52]. A research article suggested that FUS directly interacted with XRCC1 and DNA ligase III in vitro, although these interactions in the cell were mediated by PARP1 activation and PAR synthesis [34]. Both FUS and XRCC1 can interact with protein-free PAR [8,25,26,49]. It is unclear how XRCC1 can influence the FUS–PAR interactions that give rise to large assemblies. One possible explanation is that XRCC1 can be recruited into FUS–PAR assemblies and does not disrupt them. Alternatively, XRCC1 may interact with PAR and form assemblies similarly to FUS. It was therefore important to determine whether XRCC1 itself forms large assemblies in the presence of PAR. In a DLS assay, we analyzed the size of XRCC1 structures in the absence or presence of various amounts of PAR (Table 2, Figure S5). In the absence of PAR, XRCC1 itself formed only oligomers with R_h_ approximately 10 nm and was unable to form high-order assemblies (Table 3, Figure S5).

In the presence of PAR, the formation of large particles with a radius of 305 to 649 nm was observed (Table 3). Furthermore, we noticed that PAR molecules were able to modulate XRCC1 microphase separation in a concentration-dependent manner, as documented for FUS (Figure 2, Table S1). As in the case of FUS, PAR induced XRCC1 assembly in a wide range of the [XRCC1]:[ADPr] ratio of ~66:1 to 0.066:1.000, but disruption of these assemblies was seen only with a large molar excess of ADPr over XRCC1 at a [XRCC1]:[ADPr] ratio of ~1:15 to 1:30 (Table 3, Figure S5).

We also tested whether PAR exerted a similar effect on the phase behavior of YB-1, which is prone to aggregation and has PAR-binding activity (Table 3) [43,45]. The addition of various concentrations of PAR to YB-1 yielded only small particles with an R_h_ of approximately 22–26 nm (Table 3). Similar to YB-1, other PAR-binding proteins (RPA, APE1, and Pol β) were unable to form high-order assemblies in the presence of PAR (data not shown). The experiments revealed that among the tested proteins, only XRCC1 had a strong propensity for PAR-induced microphase separation under these conditions.

**Figure 7:**
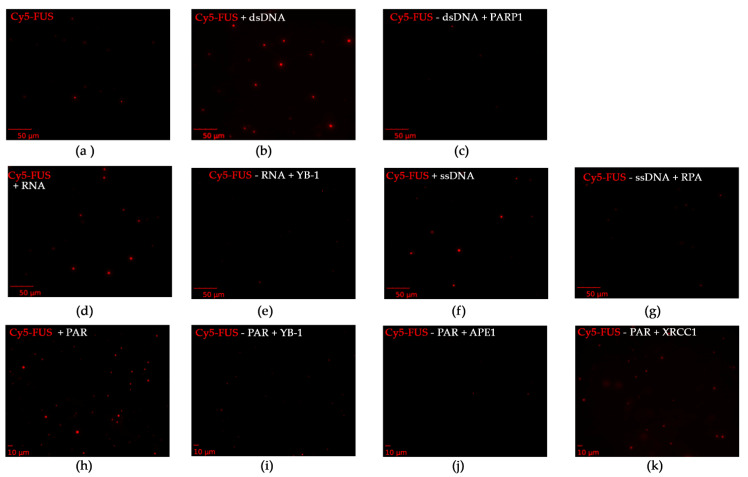
FUS–nucleic acid assemblies can be disrupted by DNA repair proteins. Fluorescence images of 4.5 µM Cy5-FUS (**a**) or 4.5 µM Cy5-FUS in the presence of 3.5 µM dNMP (dsDNA) (**b**) and 2.5 µM PARP1 (**c**); 4.5 µM Cy5-FUS in the presence of 2 µM rNMP (RNA) (**d**) and 2.8 µM YB-1 (**e**); 4.5 µM Cy5-FUS in the presence of 2.9 µM dNMP (ssDNA) (**f**) and 1.6 µM RPA (**g**); and 4.5 µM Cy5-FUS in the presence of 1 µM ADPr (PAR) (**h**) and 2.8 µM YB-1 (**i**), 3.5 µM APE1 (**j**), or 3.2 µM XRCC1 (**k**). The fluorescence photos were captured before and after the addition of a DNA repair protein to FUS–nucleic acid mixtures and 5 min incubation at room temperature.

Separately, to investigate whether these repair proteins dissolved FUS–nucleic acid assemblies, we examined FUS microdroplet assembly using fluorescence microscopy. For this purpose, we utilized Cy5-labeled FUS and assessed the stability of preformed FUS–nucleic acid assemblies under conditions in which different repair proteins were added (Figure 7). We again selected YB-1, RPA, PARP1, APE1, and XRCC1, which affected the formation of large FUS particles with R_h_ > 100 nm (Figure 4 and Figure 5). PARP1, YB-1, or RPA added at a concentration close to that of FUS was able to substantially dissolve the assemblies FUS–dsDNA, FUS–RNA, FUS–ssDNA, or FUS–PAR with only a small amount of microdroplets remaining (Figure 7c,e,g).

In the case of FUS–PAR assemblies, YB-1 or APE1 disrupted them (Figure 7i,j), whereas the addition of XRCC1 to the FUS–PAR mixture did not prevent the assembly of FUS into high-order structures (Figure 7k), implying the possibility of the assembly of XRCC1 structures with both FUS and FUS–PAR.

**Figure 8:**
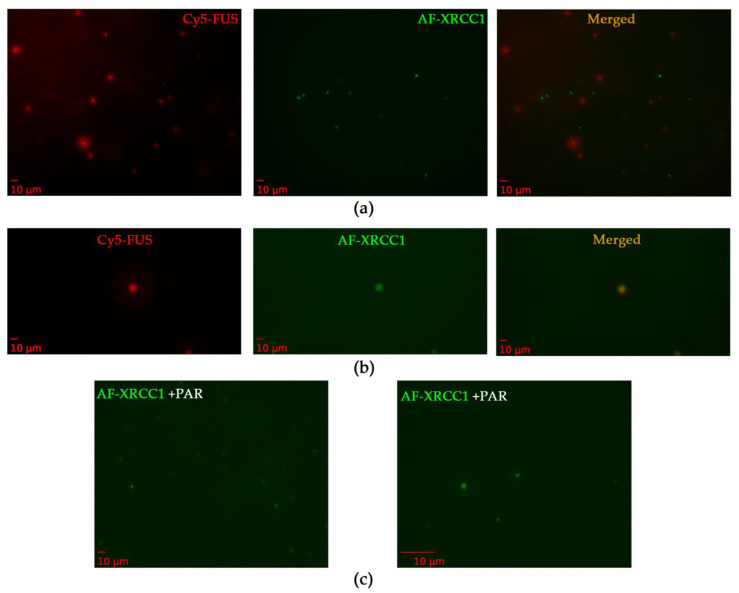
Formation of FUS and XRCC1 droplet assemblies in the presence of PAR. (**a**,**b**) Fluorescence images of 3.5 µM Cy5-FUS and 4.8 µM AF-XRCC1 in the presence of 1 µM ADPr (PAR). (**c**) Fluorescence images of 4.8 µM AF-XRCC1 in the presence of 1 µM ADPr (PAR) zoomed 20× (left panel) and zoomed 100× (right panel). Fluorescence photos were taken after the addition of PAR to FUS or XRCC1, or a FUS–XRCC1 mixture and 5 min incubation at room temperature.

XRCC1 has not been previously shown to have the ability to undergo phase separation; therefore, we investigated whether XRCC1 was concentrated into preformed FUS–PAR assemblies. For this purpose, we analyzed a mixture of Cy5-labeled FUS and AF-labeled XRCC1 to monitor the assembly of these proteins into high-order structures in the presence of PAR (Figure 8). When XRCC1 was added to preformed FUS–PAR assemblies, their coincubation produced both Cy5-FUS and AF-XRCC1 droplets (Figure 8a,b) and colocalization of Cy5 and AF signals in the individual droplets (Figure 8b).

In the absence of FUS, XRCC1 on its own formed a small number of droplets in the presence of PAR (Figure 8c). In summary, these data suggested that XRCC1 as a repair protein could colocalize with and be concentrated within FUS–PAR microphases or also form own microdroplets in the presence of PAR (Table 3, Figure 8c). Moreover, FUS and XRCC1 could compete for binding to PAR, implying similar mechanisms by which they may organize PAR/PARP1-dependent compartmentalization of damaged-DNA repair [25].

## 3. Discussion

FUS is implicated in the formation of cellular condensates linked to RNA-, PAR-, or DNA-dependent molecular events [7,8,15,16,17,34]. The characterization of the nucleic-acid-induced FUS phase separation could increase our understanding of features of the protein phase behavior and could elucidate the potential role of other proteins involved in RNA/DNA/PAR recognition and processing in the FUS-dependent organization of biomolecular condensates.

First, via DLS analysis of the hydrodynamic size of FUS and its mutants in the protein–nucleic acid mixtures, we compared the effects of the protein/nucleotide molar ratio on FUS microphase separation in an in vitro system that contained this protein or its mutant and RNA, dsDNA, ssDNA, or PAR. We observed that PAR, RNA, and DNA induced the assembly of FUS into high-order structures that was accompanied by the appearance of protein-rich microphases in a manner dependent on the nucleic acid concentration (Figure 2). 

Some authors have demonstrated that FUS LLPS in the presence of nucleic acids is reversible and highly dependent on the RNA-to-protein or ssDNA-to-protein molar ratio [17,27]. We identified the FUS-to-nucleotide ratio that led to stable assembly of FUS into high-order structures in the presence of RNA, PAR, ssDNA, or dsDNA. rNMP(RNA), dNTP(ssDNA), and dNMP(dsDNA) induced the assembly of FUS into high-order structures in a narrow range of the FUS-to-nucleotide ratio (~10:1 to 1:1; Figure 2), whereas ADPr(PAR) manifested a wider range of the ratio (~200:1 to 2:1; Figure 2). Nonetheless, the FUS–nucleic acid assemblies became unstable and dissociated when the molar concentration of nucleotides exceeded that of FUS in all cases (Figure 2). Our results showed that FUS mutations, which caused a reduction in its nucleic-acid-binding affinity and its self-assembly, strongly impaired nucleic-acid-induced assembly of FUS into high-order structures; this was particularly evident in the case of dsDNA. In the experiments with FUS and its mutants, we noted that both the C-terminal FUS RGG domain and the N-terminal FUS LCD drove the assembly of FUS into high-order structures in the presence of all types of nucleic acid polymers (Figure 3). This implied that promotion of FUS microphase separation substantially depends on the type of nucleic acid because PAR and RNA were more effective than ssDNA and dsDNA, both in the case of the wild-type FUS and of its mutants (Figure 2 and Figure 3). Accordingly, we propose that the range of the molar FUS-to-nucleotide ratio for microphase separation correlates with the affinity of FUS to a nucleic acid. Using the fluorescence titration technique, we demonstrated that FUSΔLCD (containing nucleic-acid-binding domains) possessed comparable affinities for the nucleotides ADPr and rNMP present in PAR and RNA, respectively; this affinity was in the nanomolar range (114 and 183 nM, respectively; Table 2). On the other hand, branched PAR and the long RNA transcript induced FUS microphase separation at FUS-to-nucleotide ratios differing by one order of magnitude. At the same time, the affinity for dNMP within ssDNA and dsDNA was in the micromolar range (1.1 and 2.5 µM, respectively) (Table 2), but the assembly of FUS into high-order structures began at a concentration of dNMP similar to that of rNMP (~1 µM; Table 2). According to previously published data [17,26], we believe that the nucleic-acid-induced assembly and disassembly of FUS high-order structures is affected not only by FUS’s nucleic-acid-binding affinity but also by nucleic acid structural characteristics such as length, branching, and flexibility. In any case, FUS has a clear preference for binding PAR over RNA, ssDNA, and dsDNA, as evidenced by both the protein’s affinity to PAR and by the conditions of PAR-dependent phase separation and its prolonged existence. Thus, when different nucleic acid molecules (dsDNA, RNA, and PAR) are present in the nucleus, the nucleic-acid-binding preference of FUS may play a pivotal part in the regulation of the formation of a certain type of FUS-dependent membraneless compartment. 

In our study, we also focused on the impact of individual proteins such as XRCC1, Polβ, PARP1, APE1, RPA, and YB-1 on the assembly of FUS into high-order structures in the presence of a nucleic acid. These proteins interacted with dsDNA, RNA, dsDNA, and/or PAR and participated in single-strand DNA repair (XRCC1, DNA polymerase β, PARP1, and APE1), stress-granule assembly (YB-1), and/or transcription-coupled DNA repair (RPA and PARP1); some of these proteins are members of the interactome of FUS [34,51,53,54,55,56,57]. We showed that DNA- or RNA-binding proteins including Polβ, PARP1, RPA, and YB-1 can cause dissociation of a high-order assembly of FUS whose emergence is mediated by dsDNA, ssDNA, or RNA (Figure 5 and Figure 7a-g). Similarly, these proteins—also capable of noncovalent PAR binding—affected microphase separation and destroyed a PAR-induced FUS assembly (Figure 4 and Figure 7h-j).

The important finding in our work was that XRCC1 (a PAR-binding repair factor for DNA single-strand breaks) formed own high-order assemblies and coassemblies with FUS in the presence of PAR (Table 3, Figure 8). To date, there have been no reports of XRCC1-dependent organization of biomolecular condensates in the cell, and it is possible that PAR synthesis recruits both XRCC1 and FUS to DNA damage and that repair condensates are generated with their joint participation. FUS, which is not directly involved in single-strand break repair, undergoes characteristic PAR-dependent accumulation at a DNA lesions [8,33,34]. XRCC1, which is a so-called loading platform for single-strand break repair factors, is also characterized by PAR-dependent accumulation at a DNA lesion [51,52,58,59]. In the context of DNA repair, XRCC1 alone or together with FUS may help to organize PAR-dependent DNA repair condensates. On the other hand, effective disruption of FUS–dsDNA assemblies by the presence of Polβ or PARP1 casts doubt on DNA-induced formation of a FUS condensate during base excision repair/single-strand break repair, in which both Polβ and PARP1 are active players [23,46]. Another possible scenario: the repair proteins along with nucleic acids may implement reversibility of (and control over) FUS condensation, or these specific condensates can be organized by FUS-XRCC1 cooperation. Then, PAR-dependent condensation of both FUS and DNA repair the scaffold protein—XRCC1 could play significant role in the regulation of base excision or single-strand break DNA repair. In the case of PARP1/PAR-dependent DNA repair, FUS–PAR or XRCC1-PAR interactions followed by condensate formation could be directly connected with the formation of transient repairosome compartments, which may carry out specific functions and implement spatiotemporal regulation of the DNA repair.

**Figure 9:**
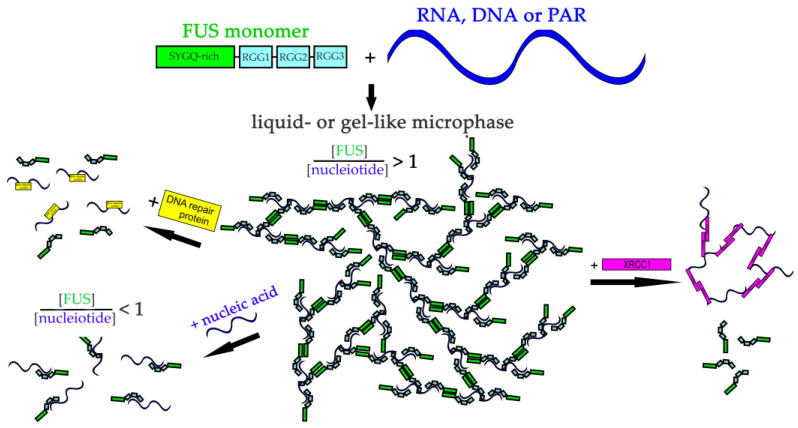
The proposed model of regulation of FUS microphase separation in the presence of nucleic acids and DNA repair proteins. RNA, DNA, and PAR could stimulate FUS phase separation. RNA/DNA/PAR-binding proteins could mediate re-entrant FUS phase separation.

Overall, our results should clarify how different nucleic acids can influence FUS phase separation and elucidate the potential function of nucleic acids and of the proteins interacting with them in the regulation of FUS-dependent condensate formation (Figure 9). 

From our findings, we can conclude that the proteins that interact with DNA, RNA, or PAR (and can disrupt a FUS–nucleic acid assembly) may modulate nucleic-acid-induced FUS microphase separation. Our data may expand the knowledge about the mechanisms governing liquid-like membraneless compartments in human cells. Further studies on how these nucleic-acid-binding proteins regulate FUS-dependent condensate formation are needed to assess the effects of both nucleic acids and nucleic-acid-binding proteins on the condensation and cellular functions of FUS.

## 4. Materials and Methods

### 4.1. Plasmids, Proteins, and Reagents

Plasmids expressing human PARP1, human apurinic/apyrimidinic endonuclease 1 (APE1), or rat DNA polymerase β (Polβ) were kindly provided by S.H. Wilson (National Institutes of Health, North Carolina, NC, USA); a human X-ray repair cross-complementing protein 1 (XRCC1) expression vector by J. P. Radicella (UMR217 CNRS/CEA, Fontenay aux Roses, France); a human Y-box-binding protein 1 (YB-1) expression vector by L.P. Ovchinnikov and D.A. Kretov (Institute of Protein Research RAS, Pushchino, Russia); and a human replication protein A (RPA) expression vector by M.S. Wold (Iowa University, USA). FUS-6E and FUS-12E expression vectors were purchased from Addgene.

Linearized plasmid pSP72−2Luc containing two full-length cDNAs of luciferases from *Renilla reniformis* and *Photinus pyralis* and serving as a template for mRNA (∼3000 nt) synthesis was kindly provided by Dmitry Lyabin (Institute of Protein Research RAS, Pushchino, Russia).

Full-length FUS(1-526), FUS∆LCD(164-526), FUS∆RGG1(1-455), FUS∆RGG1-2(1-375), FUS-6E, and FUS-12E were expressed in *Escherichia coli* strain BL21(DE3) and purified as previously described [25]. His-tagged PARP1, His-tagged XRCC1, His-tagged YB-1, APE1, Polβ, and RPA were expressed in *E. coli* strain BL21(DE3) or Rossetta(DE3) and purified as previously described [42,60,61,62,63].

Oligodeoxynucleotides were synthesized by the Laboratory of Biomedical Chemistry (ICBFM SB RAS, Novosibirsk, Russia).

Sulfo-cyanine 5 NHS ester, sulfo-cyanine 3 NHS ester, and Alexa Fluor 488 (AF) were purchased from Lumiprobe.

### 4.2. Preparation of DNA Duplexes, mRNA, and Protein-Free PAR

A DNA duplex (30 mer with a one-nucleotide gap) was obtained via hybridization of an oligonucleotide (3′-cccaaccaaacgc g taagtgtcaagaggcg-5′) with complementary oligonucleotides (5′-OH-gggttggtttgcg-3′ and 5′-phosphate-attcacagttctccgc-3′) in a 1.0:1.5 ratio. The oligonucleotide mixture was incubated for 3 min at 95 °C and then slowly cooled to room temperature. The structure of the DNA duplex is shown in Table 1.

[^32^P] labeled PAR was synthesized as described previously [64]. The size distribution of the bulk PAR was analyzed via gel electrophoresis using modified DNA sequencing gels according to [65]. The gels were dried and subjected to phosphorimaging using Typhoon FLA 7000 (GE Healthcare, United States; Figure S6). The PAR concentration was estimated via measurement of the absorbance at 258 nm (A_258_) and application of an extinction coefficient of 13.5 mM^−1^cm^−1^ for ADP-ribose (ADPr).

In vitro RNA transcript (∼3000 nt) synthesis was performed by means of the HiScribe T7 High Yield RNA Synthesis Kit (New England BioLabs). The synthesized RNA was purified using phenol extraction (Figure S7), and the RNA concentration was estimated via measurement of A_260_ with the following RNA conversion: one A_260_ unit of ssRNA = 40 μg/mL = 0.12 mM (in nucleotides).

### 4.3. Hydrodynamic Size Measurements

These measurements were performed to determine the hydrodynamic radius (R_h_) of FUS and its mutants and of the proteins RPA, YB-1, Polβ, APE1, XRCC1, and PARP1 in the presence or absence of a nucleic acid polymer.

DLS measurements were carried out using a Zetasizer Nano ZS (Malvern Instruments Ltd., Malvern, UK) at 25 °C. All stock solutions of DNA, RNA, PAR, and proteins were pre-ultrafiltered through a polyethersulfone membrane (0.2 μm pore size) in a Vivaspin centrifugal concentrator (Sartorius). The measurements and data processing were performed as described elsewhere [66].

To analyze the assembly of protein high-order structures, FUS or its mutant (FUS∆LCD, FUS∆RGG(3), FUS∆RGG(2,3), FUS-6E, or FUS-12E) was incubated in reaction mixtures composed of 10 μM protein and ADPr(PAR) (0.044–44.000 μM) or rNMP(RNA) (0.08–8.00 μM) or dNMP(ssDNA) (1.16–116.00 μM) or dNMP(dsDNA) (2.36–236.00 μM) in DLS buffer consisting of 25 mM of HEPES-NaOH pH 7.5, 200 mM of NaCl, 300 mM of urea, and 1 mM of dithiothreitol (DTT).

FUS hydrodynamic size assays in the presence of nucleic acid and nucleic-acid-binding proteins were performed in reaction mixtures consisting of (i) DLS buffer, 10 μM FUS, 0.44 μM ADPr(PAR), or 0.88 μM ADPr(PAR) and 2.4 μM YB-1 or 5.9 μM APE1 or 1.0 μM RPA or 10 μM Polβ or 2.1 μM XRCC1; (ii) 10 μM FUS, 5.5 μM dNMP(ssDNA), and 2.2 μM RPA; (iii) 10 μM FUS, 0.77 μM rNMP(RNA), and 2.0 μM YB-1; or (iv) 10 μM FUS, 7 μM dNMP(dsDNA), and 2.5 μM Polβ or 2.5 μM PARP1.

XRCC1 and YB-1 hydrodynamic size assays were performed in reaction mixtures composed of (a) DLS buffer, 2.9 μM XRCC1, and 0.044–88.000 μM ADPr (PAR); or (b) DLS buffer, 10 μM YB-1, and 0.044–44.000 μM ADPr (PAR).

The measurements were performed in a low-volume quartz batch cuvette (ZEN 2112). The samples were equilibrated for 1 min prior to the measurement. All experiments were conducted at least three times.

### 4.4. Fluorescent Labeling of FUS, FUS∆LCD, and XRCC1

For fluorescence microscopy, the proteins were labeled with a fluorescent dye (sulfo-cyanine 5 (Cy5), sulfo-cyanine 3 (Cy3), or AF) as described below. For labeling of FUS or FUS∆LCD, purified FUS (4.0 nmol) or FUS∆LCD (8 nmol) was incubated with Cy5 (34 nmol) or Cy3 (64 nmol) at 4 °C overnight in a buffer consisting of 10 mM of HEPES-NaOH pH 7.5, 50 mM of NaCl, 2.0 or 1.5 M of urea, and 100 mM of NaHCO_3_. The unreacted Cy5 or Cy3 dye was removed via dialysis against a buffer composed of 20 mM of HEPES-NaOH pH 7.5, 200 mM of NaCl, 6 M of urea, and 1 mM of DTT followed by concentration of the Cy5-labeled FUS (Cy5-FUS) or Cy3-labeled FUS∆LCD (Cy3-FUS∆LCD) in ultrafiltration spin columns.

For the labeling of XRCC1, purified XRCC1 (1.5 nmol) was incubated with AF (14 nmol) at 4 °C overnight in a buffer consisting of 20 mM of HEPES-NaOH pH 7.5 and 200 mM of NaCl. The unreacted AF dye was removed via dialysis against a buffer composed of 100 mM of sodium phosphate pH 7.2, 500 mM of NaCl, 10% glycerol, and 2 mM of DTT followed by concentrating the AF-labeled XRCC1 (XRCC1-AF) in ultrafiltration spin columns.

Concentrations of the conjugates Cy3-FUS∆LCD, Cy5-FUS, and AF-XRCC1 and the degree of labeling (DOL) were determined using the following extinction coefficients: ϵ_280_ = 70,390 M^−1^cm^−1^ for FUS, ϵ_280_ = 34,630 M^−1^cm^−1^ for FUS∆LCD, ϵ_280_ = 51,255 M^−1^cm^−1^ for XRCC1, ϵ_548_ = 162,000 M^−1^cm^−1^ and correction factor (CF_280_) 0.06 for Cy3, ϵ_495_ = 71,800 M^−1^cm^−1^ and CF_280_ = 0.1 for AF, and ϵ_646_ = 271,000 M^−1^cm^−1^ and CF_280_ = 0.04 for Cy5.

The DOL was calculated via Equation (1) using the molar extinction coefficients of the protein (ɛ_prot_) and dye (ɛ_max_), the absorbance (A_max_) at the absorption maximum of the dye, and the A_280_ of the protein:

DOL = A_max_ × ɛ_prot_/((A_280_ − A_max_ × CF_280_) × ɛ_280_) (1)

The DOL was estimated at 75% for FUS, 20% for XRCC1, and 22% for FUS∆LCD. The extinction coefficients (ɛ_prot_) of FUS, FUS∆LCD, and XRCC1 were based on the Expasy Protparam data, and the extinction coefficients (ɛ_max_) for AF, Cy5, and Cy3 were taken from Lumiprobe protocols [67].

### 4.5. Fluorescence Assays of FUS∆LCD Interaction with PAR, ssDNA, dsDNA, or RNA

Fluorescence measurements were performed at 25 °C on a POLARstar Optima multidetection microplate reader (BMG Labtech, Offenburg, Germany) in a 384-well low-volume black round-bottom polystyrene NBS microplate (Corning). The volume of the reaction mixture was 50 μL. Titration was carried out by the addition of various amounts of PAR (44–440 nM ADPr), RNA (44–440 nM rNMP), ssDNA (290–2900 nM dNMP), or dsDNA (590–5900 nM dNMP) to a fixed concentration of Cy3-FUS∆LCD (25 nM) in a buffer consisting of 25 mM of HEPES-KOH (pH 7.5), 200 mM of NaCl, 300 mM of urea, and 1 mM of DTT. For Cy3, the excitation wavelength was set to 550 nm and the emission wavelength to 570 nm.

The degree of binding (*D_b_*) and dissociation constants (*K*_d_) were estimated with the help of the following equation: D_b_ = (F − F_o_)/(F_max_ − F_0_) = 1/(1 + K_d_/[C]), where F is the measured fluorescence intensity (relative fluorescence units; RFU) of a solution containing the Cy3-FUS∆LCD conjugate at various PAR, RNA, ssDNA, or dsDNA concentrations [C]; and F_0_ and F_max_ are the fluorescence intensity in the absence and at saturating levels of the nucleic acid, respectively. All experiments were conducted at least three times.

### 4.6. Fluorescence Microscopy and Sample Preparation

Microscopy analysis of FUS or XRCC1 was carried out by means of a CELENA^®®^ S Digital Imaging System. For fluorescence microscopy, Cy5-FUS and AF-XRCC1, FAM-ssDNA, or FAM-dsDNA were used. For monitoring of FUS microdroplets, the reaction mixtures (20 µL) contained a buffer (25 mM of HEPES-NaOH pH 7.5, 200 mM of NaCl, 300 nM of urea, and 2 mM of DTT), 4.5 µM Cy5-FUS, 1 µM ADPr (PAR) or 2 µM rNMP (RNA) or 2.9 µM ssDNA or 3.5 µM dsDNA, and 2.5 µM PARP1 or 1.6 µM RPA or 2.8 µM YB-1 or 3.2 µM XRCC1 or 3.5 µM APE1, as indicated in figure legends. 

For monitoring of XRCC1 microdroplets in the presence of PAR and in the absence or presence of FUS, the reaction mixtures (20 µL) contained a buffer (25 mM of HEPES-NaOH pH 7.5, 200 mM of NaCl, and 2 mM of DTT), 0.8% PEG-20K, 4.8 µM of AF-XRCC1, 1 µM of PAR, and 3.5 µM of Cy5-FUS, as indicated in the figure legends. 

The samples were incubated for 30 min at 30 °C, after which an aliquot (6 μL) was placed between a microscopy slide and a 0.17 mm coverslip. The samples were visualized with Plan Apochromat Fluor 20 X or oil 100 X objectives with Cy5 or eGFP filters for Cy5-FUS or AF-XRCC1, respectively. Fluorescence images were captured using a Celena S Digital Imaging System.

## Figures and Tables

**Table 1:** Sequences and names of nucleic acid polymers.

Sequences	Type of Nucleic Acid	Designation
5′-gggttg gtt tgcgcattcaca gttctcg-3′	Single-stranded DNA (29 nt)	ssDNA
3′-OH 5′-phosphate \ / 5′-gggttggtttgcg attcacagttctccgc-3′ 3′-cccaaccaaacgc g taagtgtcaagaggcg-5′	Double-stranded DNA (30 bp)	dsDNA
(ADPr)_n_ (from 4 more than 30 nt)	Poly(ADP-ribose)	PAR
RNA (rNMP ~3000 nt)	RNA transcript	RNA

**Table 2:** A fluorescence-based binding assay for quantifying FUS∆LCD binding to PAR, RNA, ssDNA, or dsDNA.

Nucleotide	ADPr PAR	rNMP RNA	dNMP (ssDNA)	dNMP (dsDNA)
K_d,app_ (nM) *	114 ± 7	183 ± 11	1148 ± 68	2556 ± 159

* Cy3-labeled FUS∆LCD was titrated with various amounts of nucleic acid polymers. The data shown are mean values and standard deviations from three independent experiments. Parameters derived from a fluorescence-based binding assay as described in Section 4.5 and Figure S4.

**Table 3:** Hydrodynamic size (R_h_, nm) of XRCC1 and YB-1 at different concentrations of ADPr(PAR).

ADPr (µM)	0	0.044	0.44	4.4	44	88
XRCC1 *	10.3 ± 3.0	305 ± 41	444 ± 164	453 ± 243	29.7 ± 6.1; 649 ± 109	19.5 ± 5.0
YB-1 **	8.2 ± 1.1	8.80 ± 0.51	8.3 ± 1.8	22.6 ± 5.6	26.1 ± 8.0	

*^,^** Concentration of the protein was 2.9 μM (XRCC1) or 10 μM (YB-1). Concentration of PAR is given in nucleotide units (ADPr). The increase in the size of XRCC1 structures from ~10 to ~305–649 nm in the presence of PAR denoted the presence of large assemblies (the data highlighted in red). The data matching multimeric XRCC1–PAR or YB-1–PAR complexes are highlighted in blue.

## Supplementary Materials

The supporting information can be downloaded at: https://www.mdpi.com/article/10.3390/ijms232113200/s1.

## References

[1] Babu M.M. (2016). The contribution of intrinsically disordered regions to protein function, cellular complexity, and human disease. Biochem. Soc. Trans..

[2] Murray D.T., Kato M., Lin Y., Thurber K.R., Hung I., McKnight S.L., Tycko R. (2017). Structure of FUS protein fibrils and its relevance to self-assembly and phase separation of low-complexity domains. Cell.

[3] Law W.J., Cann K.L., Hicks G.G. (2006). TLS, EWS and TAF15: A model for transcriptional integration of gene expression. Brief Funct. Genomics.

[4] Deng H., Gao K., Jankovic J. (2014). The role of FUS gene variants in neurodegenerative diseases. Nat. Rev. Neurol..

[5] Kwon I., Kato M., Xiang S., Wu L., Theodoropoulos P., Mirzaei H., Han T., Xie S., Corden J.L., McKnight S.L. (2013). Phosphorylation-regulated binding of RNA polymerase II to fibrous polymers of low-complexity domains. Cell.

[6] Han T.W., Kato M., Xie S., Wu L.C., Mirzaei H., Pei J., Chen M., Xie Y., Allen J., Xiao G. (2012). Cell-free formation of RNA granules: Bound RNAs identify features and components of cellular assemblies. Cell.

[7] Schwartz J.C., Ebmeier C.C., Podell E.R., Heimiller J., Taatjes D.J., Cech T.R. (2012). FUS binds the CTD of RNA polymerase II and regulates its phosphorylation at Ser2. Genes Dev..

[8] Altmeyer M., Neelsen K.J., Teloni F., Pozdnyakova I., Pellegrino S., Grofte M., Rask M.B., Streicher W., Jungmichel S., Nielsen M.L. (2015). Liquid demixing of intrinsically disordered proteins is seeded by poly (ADP-ribose). Nat. Commun..

[9] Renger R., Morin J.A., Lemaitre R., Ruer-Gruss M., Jülicher F., Hermann A., Grill S.W. (2022). Co-condensation of proteins with single-and double-stranded DNA. Proc. Natl. Acad. Sci. USA.

[10] Burke K.A., Janke A.M., Rhine C.L., Fawzi N.L. (2015). Residue-by-residue view of in vitro FUS granules that bind the C-terminal domain of RNA polymerase II. Mol. Cell.

[11] Yoshimura A., Fujii R., Watanabe Y., Okabe S., Fukui K., Takumi T. (2006). Myosin-Va facilitates the accumulation of mRNA/protein complex in dendritic spines. Curr. Biol..

[12] Nishimoto Y., Nakagawa S., Hirose T., Okano H.J., Takao M., Shibata S., Suyama S., Kuwako K.I., Imai T., Murayama S. (2013). The long non-coding RNA nuclear-enriched abundant transcript 1_2 induces paraspeckle formation in the motor neuron during the early phase of amyotrophic lateral sclerosis. Mol. Brain.

[13] Sama R.R.K., Ward C.L., Kaushansky L.J., Lemay N., Ishigaki S., Urano F., Bosco D.A. (2013). FUS/TLS assembles into stress granules and is a prosurvival factor during hyperosmolar stress. J. Cell. Physiol..

[14] Patel A., Lee H.O., Jawerth L., Maharana S., Jahnel M., Hein M.Y., Stoynov S., Mahamid J., Saha S., Franzmann T.M. (2015). A liquid-to-solid phase transition of the ALS protein FUS accelerated by disease mutation. Cell.

[15] Levone B.R., Lenzken S.C., Antonaci M., Maiser A., Rapp A., Conte F., Reber S., Mechtersheimer J., Ronchi A.E., Mühlemann O. (2021). FUS-dependent liquid–liquid phase separation is important for DNA repair initiation. J. Cell Biol..

[16] Schwartz J.C., Wang X., Podell E.R., Cech T.R. (2013). RNA seeds higher-order assembly of FUS protein. Cell Rep..

[17] Maharana S., Wang J., Papadopoulos D.K., Richter D., Pozniakovsky A., Poser I., Bickle M., Rizk S., Guillén-Boixet J., Franzmann T.M. (2018). RNA buffers the phase separation behavior of prion-like RNA binding proteins. Science.

[18] Dutertre M., Lambert S., Carreira A., Amor-Guéret M., Vagner S. (2014). DNA damage: RNA-binding proteins protect from near and far. Trends Biohem. Sci..

[19] Sukhanova M.V., Singatulina A.S., Pastré D., Lavrik O.I. (2020). Fused in sarcoma (FUS) in DNA Repair: Tango with poly (ADP-ribose) polymerase 1 and compartmentalisation of damaged DNA. Int. J. Mol. Sci..

[20] Lüscher B., Ahel I., Altmeyer M., Ashworth A., Bai P., Chang P., Cohen M., Corda D., Dantzer F., Daugherty M. (2021). ADP-ribosyltransferases, an update on function and nomenclature. FEBS J..

[21] Ame J.C., Spenlehauer C., de Murcia G. (2004). The PARP superfamily. Bioessays.

[22] Alvarez-Gonzalez R., Jacobson M.K. (1987). Characterization of polymers of adenosine diphosphate ribose generated in vitro and in vivo. Biochemistry.

[23] Lavrik O.I. (2020). PARPs’ impact on base excision DNA repair. DNA Repair.

[24] Kato M., Han T.W., Xie S., Shi K., Du X., Wu L.C., Mirzaei H., Goldsmith E.J., Longgood J., Pei J. (2012). Cell-free formation of RNA granules: Low complexity sequence domains form dynamic fibers within hydrogels. Cell.

[25] Singatulina A.S., Hamon L., Sukhanova M.V., Desforges B., Joshi V., Bouhss A., Lavrik O.I., Pastré D. (2019). PARP-1 activation directs FUS to DNA damage sites to form PARG-reversible compartments enriched in damaged DNA. Cell Rep..

[26] Rhine K., Dasovich M., Yoniles J., Badiee M., Skanchy S., Ganser L.R., Ge Y., Fare C.M., Shorter J., Leung A.K. (2022). Poly (ADP-ribose) drives condensation of FUS via a transient interaction. Mol. Cell.

[27] Kang J., Lim L., Lu Y., Song J. (2019). A unified mechanism for LLPS of ALS/FTLD-causing FUS as well as its modulation by ATP and oligonucleic acids. PLoS Biol..

[28] Rhine K., Makurath M.A., Liu J., Skanchy S., Lopez C., Catalan K.F., Ma Y., Fare C.M., Shorter J., Ha T. (2020). ALS/FTLD-linked mutations in FUS glycine residues cause accelerated gelation and reduced interactions with wild-type FUS. Mol. Cell.

[29] Zinszner H., Sok J., Immanuel D., Yin Y., Ron D. (1997). TLS (FUS) binds RNA in vivo and engages in nucleo-cytoplasmic shuttling. J. Cell. Sci..

[30] Zhou Y., Liu S., Öztürk A., Hicks G.G. (2014). FUS-regulated RNA metabolism and DNA damage repair: Implications for amyotrophic lateral sclerosis and frontotemporal dementia pathogenesis. Rare Dis..

[31] Wang X., Schwartz J.C., Cech T.R. (2015). Nucleic acid-binding specificity of human FUS protein. Nucleic Acids Res..

[32] Rulten S.L., Rotheray A., Green R.L., Grundy G.J., Moore D.A., Gomez-Herreros F., Hafezparast M., Caldecott K.W. (2014). PARP-1 dependent recruitment of the amyotrophic lateral sclerosis-associated protein FUS/TLS to sites of oxidative DNA damage. Nucleic Acids Res..

[33] Mastrocola A.S., Kim S.H., Trinh A.T., Rodenkirch L.A., Tibbetts R.S. (2013). The RNA-binding protein fused in sarcoma (FUS) functions downstream of poly (ADP-ribose) polymerase (PARP) in response to DNA damage. J. Biol. Chem..

[34] Wang H., Guo W., Mitra J., Hegde P.M., Vandoorne T., Eckelmann B.J., Mitra S., Tomkinson A.E., Van Den Bosch L., Hegde M.L. (2018). Mutant FUS causes DNA ligation defects to inhibit oxidative damage repair in Amyotrophic Lateral Sclerosis. Nat. Commun..

[35] Wang W.Y., Pan L., Su S.C., Quinn E.J., Sasaki M., Jimenez J.C., Mackenzie I.R., Huang E.J., Tsai L.H. (2013). Interaction of FUS and HDAC1 regulates DNA damage response and repair in neurons. Nat. Neurosci..

[36] Niaki A.G., Sarkar J., Cai X., Rhine K., Vidaurre V., Guy B., Hurst M., Lee J.C., Koh H.R., Guo L. (2020). Loss of dynamic RNA interaction and aberrant phase separation induced by two distinct types of ALS/FTD-linked FUS mutations. Mol. Cell.

[37] Sun Z., Diaz Z., Fang X., Hart M.P., Chesi A., Shorter J., Gitler A.D. (2011). Molecular determinants and genetic modifiers of aggregation and toxicity for the ALS disease protein FUS/TLS. PLoS Biol..

[38] Wang J., Choi J.M., Holehouse A.S., Lee H.O., Zhang X., Jahnel M., Maharana S., Lemaitre R., Pozniakovsky A., Drechsel D. (2018). A molecular grammar governing the driving forces for phase separation of prion-like RNA binding proteins. Cell.

[39] Ozdilek B.A., Thompson V.F., Ahmed N.S., White C.I., Batey R.T., Schwartz J.C. (2017). Intrinsically disordered RGG/RG domains mediate degenerate specificity in RNA binding. Nucleic Acids Res..

[40] Daigle J.G., Lanson N.A., Smith R.B., Casci I., Maltare A., Monaghan J., Nichols C.D., Kryndushkin D., Shewmaker F., Pandey U.B. (2013). RNA-binding ability of FUS regulates neurodegeneration, cytoplasmic mislocalization and incorporation into stress granules associated with FUS carrying ALS-linked mutations. Hum. Mol. Genet..

[41] Monahan Z., Ryan V.H., Janke A.M., Burke K.A., Rhoads S.N., Zerze G.H., O’Meally R., Dignon G.L., Conicella A.E., Zheng W. (2017). Phosphorylation of the FUS low-complexity domain disrupts phase separation, aggregation, and toxicity. EMBO J..

[42] Henricksen L.A., Umbricht C.B., Wold M.S. (1994). Recombinant replication protein A: Expression, complex formation, and functional characterization. J. Biol. Chem..

[43] Mordovkina D., Lyabin D.N., Smolin E.A., Sogorina E.M., Ovchinnikov L.P., Eliseeva I. (2020). Y-box binding proteins in mRNP assembly, translation, and stability control. Biomolecules.

[44] Maltseva E.A., Krasikova Y.S., Sukhanova M.V., Rechkunova N.I., Lavrik O.I. (2018). Replication protein A as a modulator of the poly (ADP-ribose) polymerase 1 activity. DNA Repair.

[45] Naumenko K.N., Sukhanova M.V., Hamon L., Kurgina T.A., Anarbaev R.O., Mangerich A., Pastré D., Lavrik O.I. (2022). The C-terminal domain of Y-box-binding protein 1 exhibits structure specific binding of poly (ADP-ribose), which regulates PARP1 activity. Front. Cell Dev. Biol..

[46] Beard W.A., Wilson S.H. (2014). Structure and mechanism of DNA polymerase β. Biochemistry.

[47] Sukhanova M., Khodyreva S., Lavrik O. (2010). 2010. Poly (ADP-ribose) polymerase 1 regulates activity of DNA polymerase β in long patch base excision repair. Mutat. Res.-Fundam. Mol. Mech. Mutagen..

[48] Mok M.C., Campalans A., Pillon M.C., Guarné A., Radicella J.P., Junop M.S. (2019). Identification of an XRCC1 DNA binding activity essential for retention at sites of DNA damage. Sci. Rep..

[49] Kim I.K., Stegeman R.A., Brosey C.A., Ellenberger T. (2015). A quantitative assay reveals ligand specificity of the DNA scaffold repair protein XRCC1 and efficient disassembly of complexes of XRCC1 and the poly (ADP-ribose) polymerase 1 by poly (ADP-ribose) glycohydrolase. J. Biol. Chem..

[50] Moor N.A., Vasil’eva I.A., Kuznetsov N.A., Lavrik O.I. (2020). Human apurinic/apyrimidinic endonuclease 1 is modified in vitro by poly (ADP-ribose) polymerase 1 under control of the structure of damaged DNA. Biochimie.

[51] Caldecott K.W. (2019). XRCC1 protein; Form and function. DNA Repair.

[52] Hanzlikova H., Gittens W., Krejcikova K., Zeng Z., Caldecott K.W. (2017). Overlapping roles for PARP1 and PARP2 in the recruitment of endogenous XRCC1 and PNKP into oxidized chromatin. Nucleic Acids Res..

[53] Caldecott K.W. (2001). Mammalian DNA single-strand break repair: An X-ra(y)ted affair. BioEssays.

[54] Hamon L., Budkina K., Pastré D. (2022). YB-1 Structure/Function Relationship in the Packaging of mRNPs and Consequences for Translation Regulation and Stress Granule Assembly in Cells. Biochemistry (Moscow).

[55] Marteijn J.A., Lans H., Vermeulen W., Hoeijmakers J.H. (2014). Understanding nucleotide excision repair and its roles in cancer and ageing. Nat. Rev. Mol. Cell Biol..

[56] Reber S., Jutzi D., Lindsay H., Devoy A., Mechtersheimer J., Levone B.R., Domanski M., Bentmann E., Dormann D., Mühlemann O. (2021). The phase separation-dependent FUS interactome reveals nuclear and cytoplasmic function of liquid–liquid phase separation. Nucleic Acids Res..

[57] Tosolini D., Antoniali G., Dalla E., Tell G. (2020). Role of phase partitioning in coordinating DNA damage response: Focus on the Apurinic Apyrimidinic Endonuclease 1 interactome. Biomol. Concepts.

[58] Wei L., Nakajima S., Hsieh C.L., Kanno S., Masutani M., Levine A.S., Yasui A., Lan L. (2013). Damage response of XRCC1 at sites of DNA single strand breaks is regulated by phosphorylation and ubiquitylation after degradation of poly(ADP-ribose). J. Cell. Sci..

[59] Breslin C., Hornyak P., Ridley A., Rulten S.L., Hanzlikova H., Oliver A.W., Caldecott K.W. (2015). The XRCC1 phosphate-binding pocket binds poly (ADP-ribose) and is required for XRCC1 function. Nucleic Acids Res..

[60] Sukhanova M.V., Khodyreva S.N., Lavrik O.I. (2004). Poly(ADP-ribose) polymerase-1 inhibits strand-displacement synthesis of DNA catalyzed by DNA polymerase β. Biochemistry.

[61] Belousova E.A., Vasil’eva I.A., Moor N.A., Zatsepin T.S., Oretskaya T.S., Lavrik O.I. (2013). Clustered DNA lesions containing 5-formyluracil and AP site: Repair via the BER system. PLoS ONE.

[62] Lebedeva N.A., Khodyreva S.N., Favre A., Lavrik O.I. (2003). AP endonuclease 1 has no biologically significant 3′→ 5′-exonuclease activity. Biochem. Biophys. Res. Commun..

[63] Drachkova I.A., Petruseva I.O., Safronov I.V., Zakharenko A.I., Shishkin G.V., Lavrik O.I., Khodyreva S.N. (2001). Reagents for modification of protein–nucleic acids complexes with primers elongated by the dCTP exo-N-substituted arylazido derivatives. Russ. J. Bioorg. Chem..

[64] Amé J.C., Héberlé É., Camuzeaux B., Dantzer F., Schreiber V. (2017). Purification of recombinant human PARG and activity assays. Methods Mol. Biol..

[65] Panzeter P.L., Althaus F.R. (1990). High resolution size analysis of ADP-ribose polymers using modified DNA sequencing gels. Nucleic Acids Res..

[66] Vasil’eva I.A., Anarbaev R.O., Moor N.A., Lavrik O.I. (2019). Dynamic light scattering study of base excision DNA repair proteins and their complexes. Biochim. Biophys. Acta.

[67] Lumiprobe Life Science Solutions. https://www.lumiprobe.com.

