# Supplementary material for: FUS Microphase Separation: Regulation by Nucleic Acid Polymers and DNA Repair Proteins
Source: Int J Mol Sci. 2022 Oct 30;23(21):13200. doi: 10.3390/ijms232113200 (PMC9654039; doi:10.3390/ijms232113200)
Supplement: Supplementary file 1 [file ijms-23-13200-s001.zip › ijms-1930320-supplementary.pdf]

Supplementary Materials

1. Supplementary Tables

Table S1. The hydrodynamic radius (R_h, nm) for FUS as determined in the presence of different concentrations of RNA(DNA) nucleotides and ADPr.

Nucleotide*		ADPr(PAR)				rNMP(RNA)			dNMP(ssDNA)			dNMP(dsDNA)		
μM	0	0.044	0.44	4.4	44	0.08	0.8	8.0	1.16	11.6	116	2.36	23.6	236
FUS	3.67	119	150	225	31.3	3.39	129	171	138	215	3.77	153	158	2.78
	±0.35	±21	±31	±62	±3.9	±0.43	±30	±30	±29	±78	±0.65	±27	±18	±0.53

* Molar concentration of nucleic acid polymers is presented in nucleotide units (ADPr, rNTP, or dNTP). The increase in the size of FUS structures from ~4 to ~119–225 nm in the presence of a nucleic acid polymer denotes the presence of large assemblies (the data highlighted in red). Subsequent incubation of FUS with higher concentrations of nucleic acids reduces the large assemblies to monomers of FUS or FUS–nucleic acid complexes (the data highlighted in blue).

Table S2. The hydrodynamic radius (Rh, nm) for FUS-6E or FUS-12E as determined in the presence of different concentrations of RNA(DNA) nucleotides and ADPr.

Nucleotide *		ADPr(PAR)				rNMP(RNA)			dNMP(ssDNA)			dNMP(dsDNA)		
μM	0	0.044	0.44	4.4	44	0.08	0.8	8.0	1.16	11.6	116	2.36	23.6	236
FUS -6E	3.99	3.85	148	280	4.9	3.83	130	11.1	3.78	4.27	3.43	4.24	3.90	3.22
	±0.28	±0.17	±28	±46	±1.5	±0.20	±9	±3.4	±0.53	±0.35	±0.67	±0.13	±0.30	±0.34
FUS-12E	4.01	3.98	4.15	5.07	6.01	3.91	3.82	4.13	4.23	4.45	4.66	3.85	4.15	3.53
	±0.31	±0.35	± 0.26	±0.88	±0.53	±0.25	±0.38	±0.10	±0.27	±0.56	±0.47	±0.30	±0.15	±0.38

* Molar concentration of nucleic acid polymers is presented in nucleotide units (ADPr, rNTP, or dNTP). The increase in the size of FUS-6E or FUS-12E structures from ~4 to ~130–280 nm in the presence of a nucleic acid polymer denotes the presence of large assemblies (the data highlighted in red). Subsequent incubation of FUS with higher concentrations of nucleic acids reduces the large assemblies to monomers of FUS or FUS–nucleic acid complexes (the data highlighted by blue).

Table S3. The hydrodynamic radius (R_h, nm) for FUSΔRGG(3) and FUSΔRGG(2,3) as determined in the presence of different concentrations of RNA(DNA) nucleotides and ADPr.

Nucleotide *		ADPr(PAR)				rNMP(RNA)			dNMP(ssDNA)			dNMP(dsDNA)		
μM	0	0.044	0.44	4.4	44	0.08	0.8	8.0	1.16	11.6	116	2.36	23.6	236
FUS ΔRGG(3)	4.23	3.77	394	362	553	3.8	141	392	3.80	163	4.36	4.10	3.83	4.15
	±0.31	±0.32	±129	±42	±55	±0.60	±38	±33	±0.29	±30	±0.10	±0.13	±0.24	±0.20
FUS ΔRGG (2,3)	4.23	3.57	3.64	3.63	3.80	3.68	3.95	3.61	4.17	4.03	3.79	4.19	4.27	3.71
	±0.44	±0.19	±0.15	±0.10	±0.20	±0.22	±0.20	±0.37	±0.42	±0.16	±0.10	±0.14	±0.16	±0.29

* Molar concentration of nucleic acid polymers is presented in nucleotide units (ADPr, rNTP, or dNTP). The increase in the size of FUSΔRGG(3) and FUSΔRGG(2,3) from ~4 to ~163–392 nm in the presence of a nucleic acid polymer indicates the presence of large assemblies (the data highlighted in red). Subsequent incubation of FUS with higher concentrations of nucleic acids reduces the large assemblies to monomers of FUS.

2. Supplementary Figures

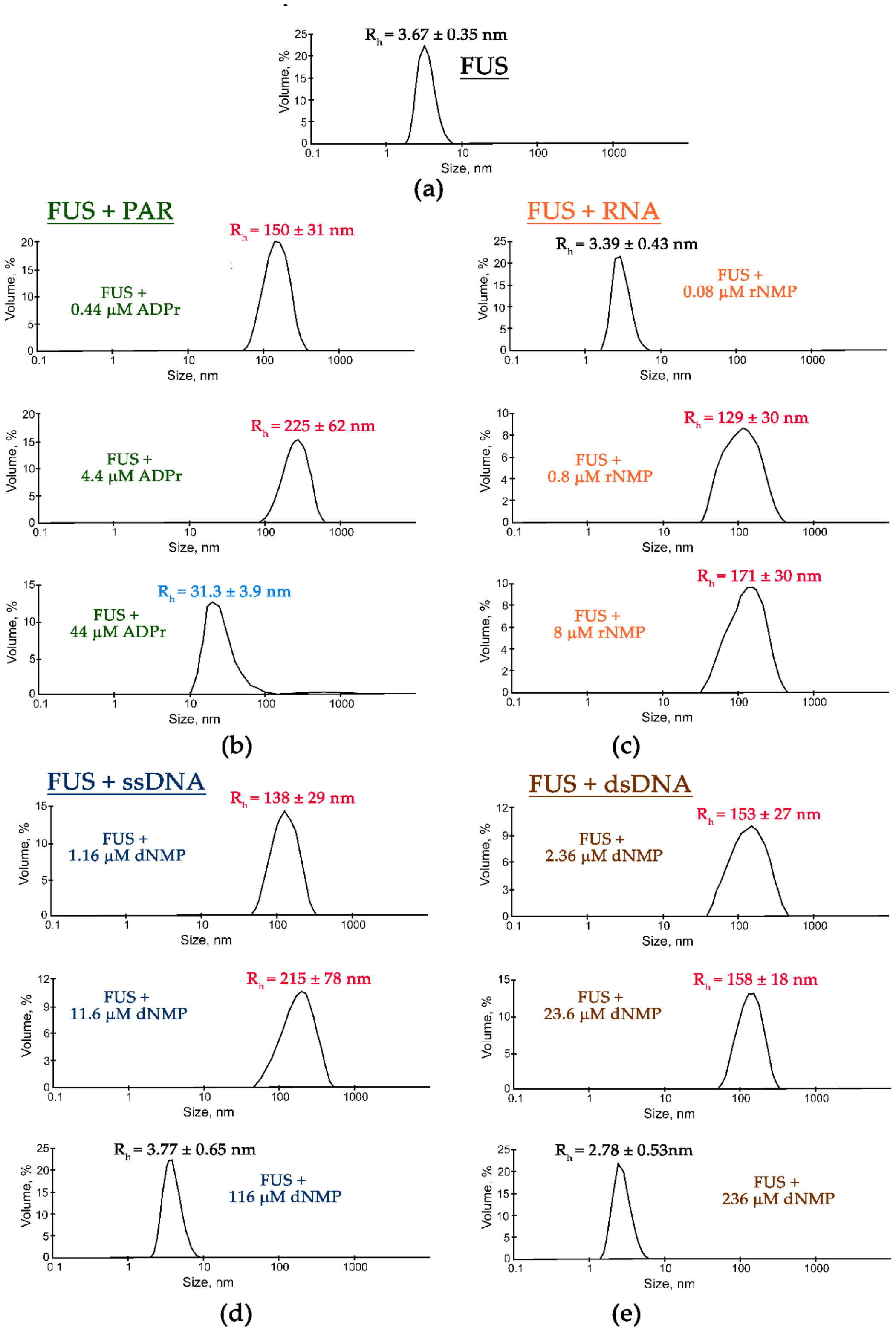

Figure S1: Typical volume-weighted size distributions for FUS (a), a FUS – PAR (b), FUS -RNA (c), FUS - ssDNA (d) or FUS – dsDNA (e) mixtures. The profiles were obtained by means of experimental autocorrelation functions in the Zetasizer Nano ZS software. The average hydrodynamic radii (R_h) computed from the distributions are presented as well. R_h is the average R_h value estimated from at least three DLS experiments. FUS high-order structure assays in the presence of nucleic acid polymer were performed in reaction mixtures consisting of 10 µM FUS and ADPr(PAR) (0.044–44.000 µM) or rNMP(RNA) (0.08–8.00 µM) or dNMP(ssDNA) (1.16–116.00 µM) or dNMP(dsDNA) (2.36–236.00 µM) in

DLS buffer consisting of 25 mM HEPES-NaOH pH 7.5, 200 mM NaCl, 300 mM urea, and 1 mM dithiothreitol (DTT). The R_h values were measured directly after 1-min incubation of FUS with nucleic acid.

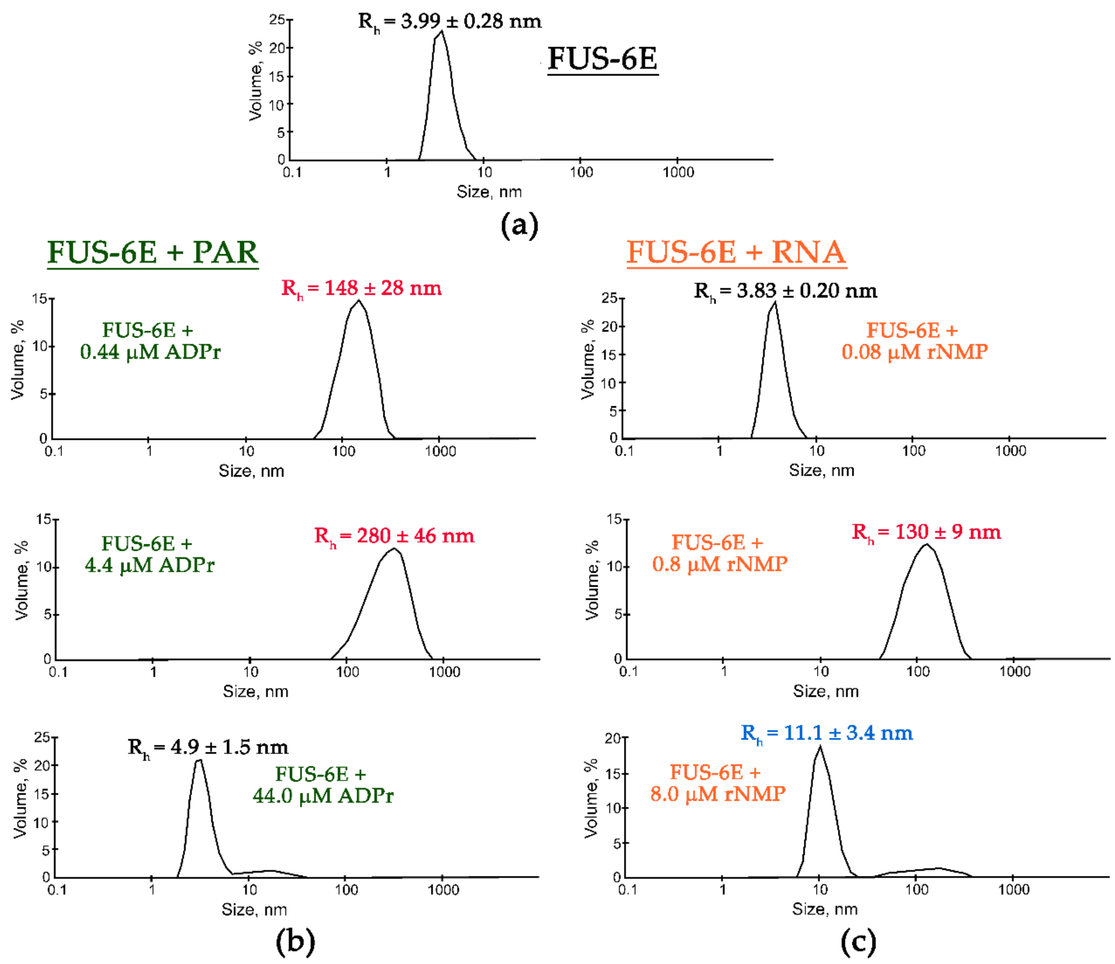

Figure S2: Typical volume-weighted size distributions for FUS-6E (a) and a FUS-6E – PAR (b) and FUS-6E - RNA (c) mixtures. The profiles were obtained by means of experimental autocorrelation functions in the Zetasizer Nano ZS software. The average hydrodynamic radii (Rh) computed from the distributions are presented as well. R_h is the average R_h value estimated from at least three DLS experiments.

FUS-6E high-order structure assays in the presence of nucleic acid polymer were performed in reaction mixtures consisting of 5 μM FUS-6E and ADPr(PAR) (0.044–44.000 μM) or rNMP(RNA) (0.08–8.00 μM) in DLS buffer consisting of 25 mM HEPES-NaOH pH 7.5, 200 mM NaCl, 300 mM urea, and 1 mM DTT. The R_h values were measured directly after 1-min incubation of FUS-6E with PAR or RNA.

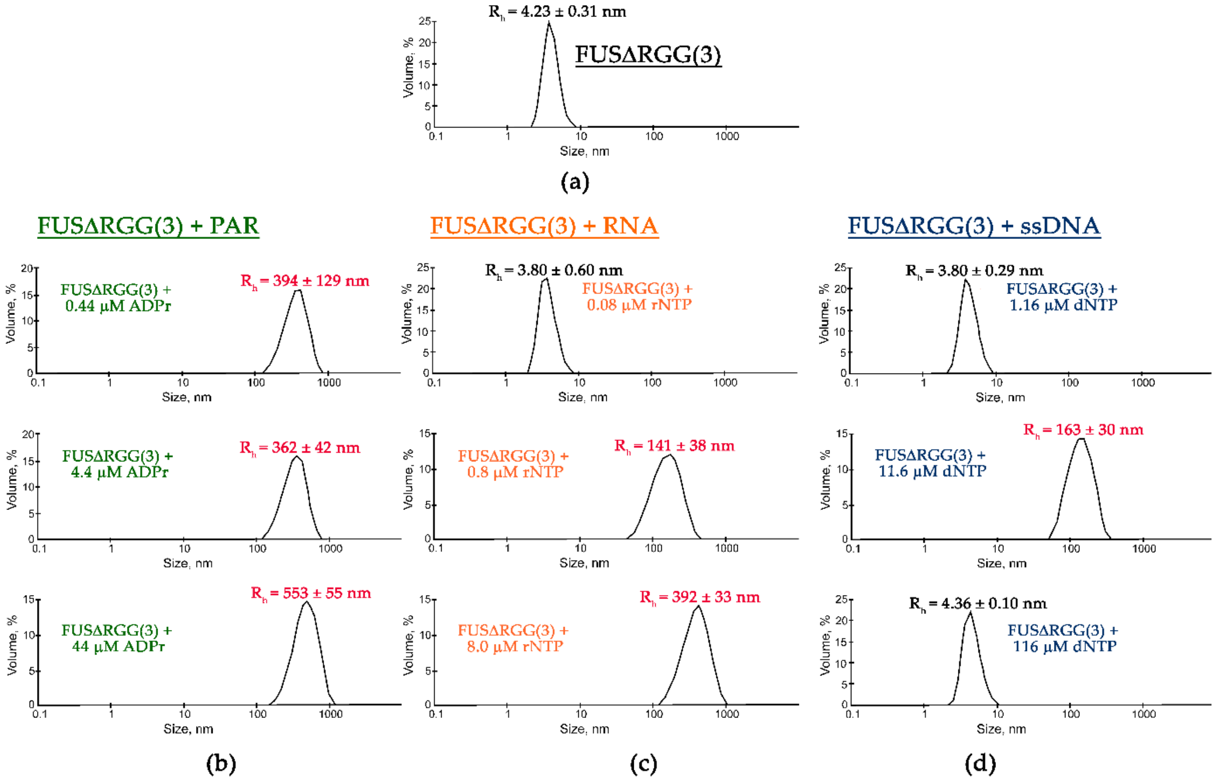

Figure S3: Typical volume-weighted size distributions for FUSΔRGG(3) (a) and a FUSΔRGG(3) – PAR (b) and FUSΔRGG(3) – RNA (c) and FUSΔRGG(3) – ssDNA (d) mixtures. The profiles were obtained by means of experimental autocorrelation functions in the Zetasizer Nano ZS software. The average hydrodynamic radii (Rh) computed from the distributions are presented as well. R_h is the average R_h value estimated from at least three DLS experiments. FUSΔRGG(3) high-order structure assays in the presence of nucleic acid polymer were performed in reaction mixtures consisting of 5 μM FUSΔRGG(3) and ADPr(PAR) (0.044–44.000 μM) or rNMP(RNA) (0.08–8.00 μM) or dNMP(ssDNA) (1.16–116.00 μM) in DLS buffer consisting of 25 mM HEPES-NaOH pH 7.5, 200 mM NaCl, 300 mM urea, and 1 mM DTT. The R_h values were measured directly after 1-min incubation of FUSΔRGG(3) with PAR or RNA or ssDNA.

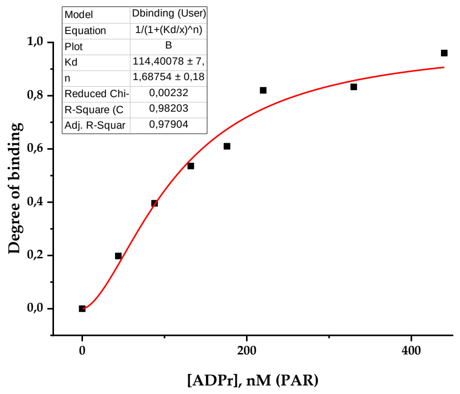

(**a**)

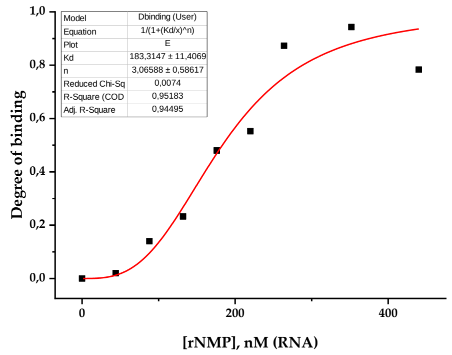

(**b**)

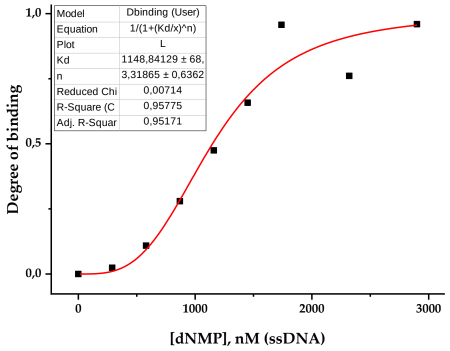

(**c**)

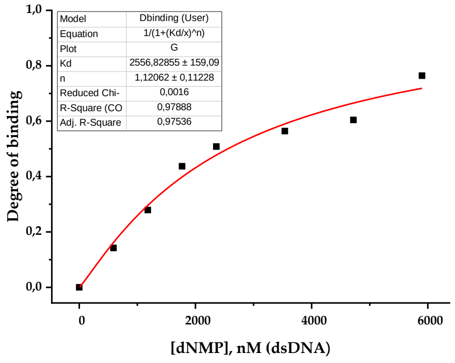

(**d**)

Figure S4: A fluorescence-based binding assay for quantifying FUSΔLCD binding to PAR (a), RNA (b), ssDNA (c) or dsDNA (d). A Cy3-labeled FUSΔLCD was titrated with various amounts of nucleic acid polymers. Titration was carried out by the addition of various amounts of PAR (44–440 nM ADPr), RNA (44–440 nM rNMP), ssDNA (290–2900 nM dNMP), or dsDNA (590–5900 nM dNMP) to a fixed concentration of Cy3-FUSΔLCD (25 nM) in a buffer consisting of 25 mM HEPES-KOH (pH 7.5), 200 mM NaCl, 300 mM urea, and 1 mM DTT.

Graphs show titration curves that are representative of mean values of three independent experiments. Curves show best fits (R^2 values met or exceeded 0.95) of following equation: $D_b = (F \quad F_o)/(F_{max} \quad F_0) = 1/(1 + K_d/[C])$, where D_b is degree of binding, F is the measured fluorescence intensity (relative fluorescence units; RFU) of a solution containing the Cy3-FUSΔLCD conjugate at various nucleotide concentrations [C], F_0 and F_{max} are fluorescence intensity in the absence of and at saturating levels of the nucleic acid, respectively.

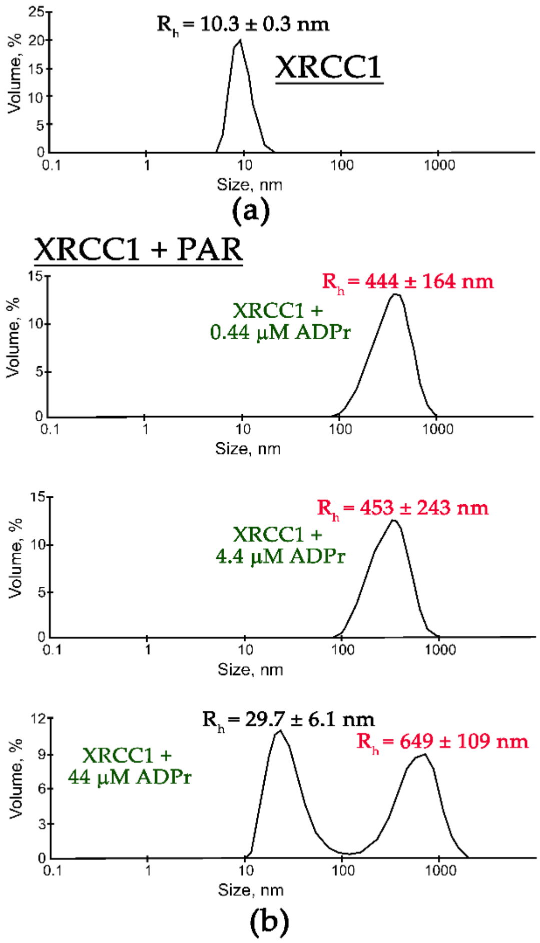

Figure S5: Typical volume-weighted size distributions for XRCC1 (a) and a XRCC1 – PAR (b) mixtures. The profiles were obtained by means of experimental autocorrelation functions in the Zetasizer Nano ZS software. The average hydrodynamic radii (R_h) computed from the distributions are presented as well. R_h is the average R_h value estimated from at least three DLS experiments. XRCC1 high-order structure assays in the presence of nucleic acid polymer were performed in reaction mixtures consisting of 2.9 µM XRCC1 and ADPr(PAR) (0.044–88.000 µM) in DLS buffer consisting of 25 mM HEPES-NaOH pH 7.5, 200 mM NaCl, 300 mM urea, and 1 mM DTT. The R_h values were measured directly after 1-min incubation of XRCC1 with PAR.

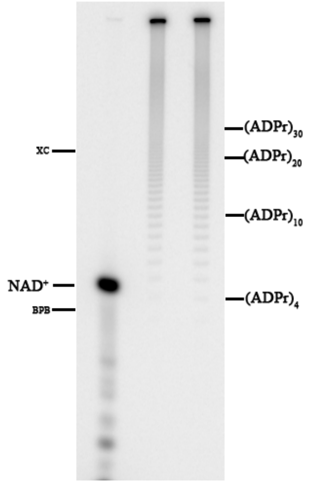

Figure S6: Size-distribution of bulk ADP-ribose polymers synthesized by PARP1. The reaction mixtures (500 µl) contained 50 mM Tris-HCl, pH 8.0, 4 mM $MgCl_2$, 50 µg/ml BSA, 600 nM DNA (30-bp), 35 nM PARP1, 500 mM NAD+ + 0.01 µCi $[^{32}P]$NAD+, and 5 mM $MgCl_2$. The reactions were incubated for 1 h at 37°C. After that, bulk PAR was purified as described early (Amé et al., *Methods Mol. Biol*. **2017**). The PAR were analyzed by gel-electrophoresis using "modified DNA sequencing gels" according to Panzeter, P. L. and Althaus, F. R. (*Nucleic Acids Res.* **1990**). The gels were dried and subjected to phosphorimaging using Molecular Imager/Quantity One software (Bio-Rad, USA**).**

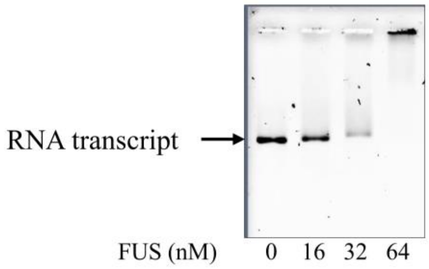

Figure S7: Analysis of RNA transcript. RNA (0.8 nM) was incubated with FUS in buffer consisting of 12.5 mM Hepes, pH=8.0, 25 mM NaCl, 260 mM Urea for 10 min at 37°C. Analysis of RNA transcript and RNA-FUS complexes was done by electrophoresis on 0.8% agarose gel in 0.5 X TBE buffer. The gel was stained with GelRed (Biotium, USA) and imaged on Typhoon FLA 9500 for visualization using ethidium bromide filter.
